# Development of a Novel Covalently Bonded Conjugate of Caprylic Acid Tripeptide (Isoleucine–Leucine–Aspartic Acid) for Wound-Compatible and Injectable Hydrogel to Accelerate Healing

**DOI:** 10.3390/biom14010094

**Published:** 2024-01-11

**Authors:** Sachin B. Baravkar, Yan Lu, Abdul-Razak Masoud, Qi Zhao, Jibao He, Song Hong

**Affiliations:** 1 Neuroscience Center of Excellence, School of Medicine, L.S.U. Health, New Orleans, LA 70112, USA; 2 NMR Laboratory, Department of Chemistry, Tulane University, New Orleans, LA 70118, USA; 3 Microscopy Laboratory, Tulane University, New Orleans, LA 70118, USA; 4 Department of Ophthalmology, School of Medicine, L.S.U. Health, New Orleans, LA 70112, USA

**Keywords:** caprylic acid, tripeptide, ultrashort peptide, covalently bonded conjugate, injectable, amphiphile, hydrogel, skin third-degree burn, wound healing, sheer thinning, re-gelation, hydrogelable, hydrogelability, wound closure, re-epithelialization, fmoc/tBu solid-phase peptide synthesis, rational design, molecular template, modulus, viscoelasticity, storage modulus, loss modulus, thixotropy, rheology, flow point, gel state, sol state

## Abstract

Third-degree burn injuries pose a significant health threat. Safer, easier-to-use, and more effective techniques are urgently needed for their treatment. We hypothesized that covalently bonded conjugates of fatty acids and tripeptides can form wound-compatible hydrogels that can accelerate healing. We first designed conjugated structures as fatty acid–aminoacid1–amonoacid2–aspartate amphiphiles (Cn acid–AA1–AA2–D), which were potentially capable of self-assembling into hydrogels according to the structure and properties of each moiety. We then generated 14 novel conjugates based on this design by using two Fmoc/tBu solid-phase peptide synthesis techniques; we verified their structures and purities through liquid chromatography with tandem mass spectrometry and nuclear magnetic resonance spectroscopy. Of them, 13 conjugates formed hydrogels at low concentrations (≥0.25% *w*/*v*), but C8 acid-ILD-NH_2_ showed the best hydrogelation and was investigated further. Scanning electron microscopy revealed that C8 acid-ILD-NH_2_ formed fibrous network structures and rapidly formed hydrogels that were stable in phosphate-buffered saline (pH 2–8, 37 °C), a typical pathophysiological condition. Injection and rheological studies revealed that the hydrogels manifested important wound treatment properties, including injectability, shear thinning, rapid re-gelation, and wound-compatible mechanics (e.g., moduli G″ and G′, ~0.5–15 kPa). The C8 acid-ILD-NH_2_(**2**) hydrogel markedly accelerated the healing of third-degree burn wounds on *C57BL/6J* mice. Taken together, our findings demonstrated the potential of the Cn fatty acid–AA1–AA2–D molecular template to form hydrogels capable of promoting the wound healing of third-degree burns.

## 1. Introduction

Third-degree burn injuries involve the destruction of full-thickness skin, posing a significant health threat [1,2,3]. The typical treatment for burn wounds involves debridement, followed by the application of topical and antimicrobial agents to support skin rebuilding and vascular network formation [4]. In general, the traditional clinical methods to treat wounds, including third-degree burns, are anti-infection, vacuum suction, oxygen therapy, and the use of dressings such as gauze, foams, bandages, hydrocolloids, iodine and silver dressings, and films. Some third-degree burn wounds are also repaired using skin grafts by harvesting healthy skin from other parts of the body. However, graft harvesting itself results in new wounds, thereby compromising the function of the donor sites. The overall graft procedure is lengthy, risky, and costly and can result in debilitation, thereby necessitating the development of a safer, easier-to-use, and more effective technique for the repair of third-degree burns.

The most important factor for the rapid healing of wounds is the maintenance of a moist environment, as this can facilitate high oxygen permeation and wound exudate removal. Therefore, therapeutics should promote these processes while also supporting proliferation and tissue remodeling [5]. Naturally occurring polymers, such as chitosan, alginate, elastin, cellulose, fibrin, hyaluronic acid, pectin, dextran, and collagen, are biopolymers that are generally used in wound dressings [6,7,8,9,10,11,12,13,14]. However, these natural polymers have poorer stability, structural heterogeneity, and mechanical properties than their synthetic counterparts, which also possess other merits, such as the ease of application, appropriate firmness and elasticity, and superior water retention capacity [15]. Therefore, biomaterials are usually incorporated into hydrogels for wound dressings, and various polymer-based hydrogels, such as polyethylene glycol or polyvinyl alcohol, can serve as inert supports/scaffolds [16]. Hydrogels are used to promote wound healing because they can hold large amounts of water or biological fluid, thereby structurally mimicking the three-dimensional (3D) network structure of the natural extracellular matrix [17,18,19,20,21,22,23]. Hydrogels fabricated from natural tissue components, such as collagens, fibrins, and cellular dermal matrices, have been used successfully to promote wound repair and regeneration [24,25,26]; however, tissue-derived natural biomaterials have several disadvantages, including high cost, poor reproducibility, low availability, and the potential risk of disease transmission [24,25,26]. To overcome these shortcomings, synthetic biomaterials that can be fabricated into hydrogels are highly desirable, as they can mimic the structural, mechanical, and chemical properties of skin.

Peptide-based hydrogels generated through organic synthesis are inherently biocompatible and biodegradable because they can be broken down to individual nutrient amino acids by enzymes present in tissues [27]. Concomitantly, peptide-incorporated hydrogels have found enormous applications in the biochemical and biomedical fields as materials for the sustained release of drugs and biomolecules [28], cell culture substrates, tissue engineering [29], and (pertinent to this report) scaffolds for wound healing [30,31]. Peptides containing aromatic amino acids, including NFGAIL [32], DFNKF [33], KLVFFAE [34], and FDFSFDFS [35], tend to assume a β-sheet conformation due to π−π stacking that drives fibril formation. The pentapeptide KYFIL forms a stimulus-responsive and highly stable injectable hydrogel for tissue engineering applications [36]. Similarly, an ultrashort peptide-based hydrogel showing high mechanical properties has been used for the healing of critical bone defects in rabbits [37]. Peptide amphiphiles containing a hydrophobic alkyl tail and a hydrophilic peptide domain, such as LIVAGD, can spontaneously self-assemble into hydrogels in an aqueous solution to form robust hydrogels via β-sheet assembly [38].

We hypothesized that a covalently bonded conjugate of a fatty acid and tripeptide would form a wound-compatible hydrogel capable of accelerating healing. We aimed to develop a hydrogelator that is as simple and as small as the previously reported ultrashort peptide hydrogelators, easily made, and effective in promoting the healing of third-degree burns. Its hydrogel should have the following properties: be compatible with burn wounds in terms of viscoelasticity (0.5–15 kPa [39,40]), injectable to ensure easy application to wounds, stable on wounds for more than a week (with a pH of 2–8); have the osmolarity of saline; exert no systematic or local toxicity; and should be fully degradable when the wounds are healed. The advantages of using fatty acids are that they are safe as they naturally exist in the human body, can be easily linked to the N-terminal amino groups of peptides via amide bonds, and can participate in hydrogelation through their hydrophobic carbon chains as a moiety of the hydrogelator. To the best of our knowledge, no hydrogelator with this composition has yet been reported. Based on our previous unreported trials, we targeted a tripeptide as a moiety of the hydrogelator. Additionally, longer peptides have more amide bonds, which may not only increase the preparation cost but also increase the sites for undesired enzymatic degradation or nonenzymatic hydrolysis of the peptides, thus impacting the stability of the peptides. Shorter peptides (dipeptides) may have lower gelability than longer peptides because of the lower intermolecular interaction (hydrogen bonds and hydrophobic force) [32,33,34,35].

Here, we report our approaches to testing our hypothesis and achieving our objectives. We have developed novel covalently bonded conjugates of caprylic acid–tripeptide (isoleucine–leucine–aspartic acid) compounds capable of hydrogelation. These compounds were rationally designed and synthesized using Fmoc/tBu-based solid-phase peptide synthesis (SPPS) strategies and characterized using liquid chromatography with tandem mass spectrometry (LC-MS/MS) and NMR spectroscopic techniques. The hydrogelation was undertaken in saline, followed by concentration and pH studies, and the swelling ratio (%) for a representative compound (**2**) was obtained. The novel hydrogel from (**2**) has proven injectable properties and its mechanical properties revealed that **2** possesses a wound-compatible storage modulus and excellent shear-thinning ability. Compound **2** can form a wound-compatible and injectable hydrogel capable of accelerating the wound healing of third-degree burn wounds.

## 2. Materials and Methods

### 2.1. Materials

Fmoc-protected amino acids—Fmoc-Ile-OH (CAS No. 71989-23-6, 98%), Fmoc-Ala-OH (CAS No. 35661-39-3, 99.92%), Fmoc-Val-OH (CAS No. 68858-20-8, 99.56%), Fmoc-Leu-OH (CAS No. 35661-60-0, 99.78%), and Fmoc-Asp(OtBu)-OH (CAS No. 71989-14-5, 99.92%)—and fatty acids, namely n-octanoic acid (CAS No. 124-07-2, 99.56%) and tetradecanoic acid (CAS No. 544-63-8, 99%), were purchased from BLD Pharmatech (Cincinnati, OH, USA) and used without further purification. Lauric acid (CAS No. 143-07-7) and palmitic acid (CAS No. 57-10-3, 99%) were purchased from Sigma Chemicals (St. Louis, MO, USA). Fmoc-rink amide resin (0.57 mmol/g, 100–200 mesh); Wang resin (0.9 mmol/g, 100–200 mesh); the Kaiser test kit (Catalog no. KGZ001); O-benzotriazole-N, N, N′; and N′-tetramethyluronium-hexafluoro-phosphate (CAS No. 94790-37-1) were purchased from Aapptec, LLC (Louisville, KY, USA). Hydroxybenzotriazole (HOBt, CAS No. 2592-95-2, 98.75%) was purchased from Apexbio (Houston, TX, USA). Trifluoroacetic acid (TFA; CAS No. 76-05-1, 99%) was purchased from Honeywell Research Chemicals (Muskegon, MI, USA). Piperidine (CAS No. 110-89-4, 99%) was purchased from Sigma-Aldrich LLC (St. Louis, MI, USA). N, N-diisopropylethylamine (DIPEA; CAS No. 7087-68-5, 99%) was purchased from TCI America (Portland, OR, USA). N, N-dimethylformamide (DMF; CAS No. 68-12-2), diethyl ether (DE; CAS No. 60-29-7), and dichloromethane (DCM; CAS No. 75-09-2) were obtained from Thermo-Scientific (Ward Hill, MA, USA).

### 2.2. Organic Synthesis and Structure Verification of Fatty Acid-Conjugated Tripeptides

The fatty acid-conjugated peptides were synthesized manually using Fmoc/tBu-based SPPS strategies using PolyPrep columns obtained from Bio-Rad Laboratories (Hercules, CA, USA). The synthesis was conducted on a 0.1 mmol scale on the Fmoc-Rink amide resin (for the C-terminus amide) and Wang resin (for the C-terminus acid).

#### 2.2.1. General Procedure for Synthesis of C-Terminus Amide Conjugates

The Fmoc-Rink amide resin (175 mg) was swollen in DCM (2.0 mL) in a Bio-Rad column for 30 min. The solvent was then pushed out with positive pressure and replaced with 20% piperidine in DMF (2.0 mL), and the mixture was shaken for 20 min. The solvent was removed, and the resin was washed with DMF (3 × 2 mL) and DCM (3 × 2 mL) (confirmed by a positive Kaiser test). The Fmoc-Asp(tBu)-OH (164 mg) was placed in a scintillation vial and dissolved in DMF (2.0 mL) together with HBTU (152 mg), HOBt (54 mg), and DIPEA (0.2 mL). The mixture was sonicated for 1 min and then added to a resin column, which was shaken on a vortex mixer for 6 h. The solvent was removed from the column, and the column was washed with DMF (3 × 2 mL) and DCM (3 × 2 mL) (confirmed by a negative Kaiser test). The resin was endcapped by adding 5 mL of acetic anhydride/pyridine solution (3:2, *v*/*v*) and rocking the resin for 1 h, followed by washing with DMF (3 × 2 mL) and DCM (3 × 2 mL) (confirmed by a negative Kaiser test).

The Fmoc group was removed by adding 20% piperidine in DMF (2.0 mL) and shaking for 20 min. The solvent was removed, and the column was washed with DMF (3 × 2 mL) and DCM (3 × 2 mL) (confirmed by a positive Kaiser test). In a 10 mL scintillation vial, the second amino acid (4 equiv), HBTU (152 mg), HOBt (54 mg), and DIPEA (0.2 mL) were dissolved in DMF (2.0 mL). This mixture was shaken well for 10 min and added to the column, and the column was vortexed for 6 h. The solvent was removed from the column, and the column was washed with DMF (3 × 2 mL) and DCM (3 × 2 mL) (confirmed by a negative Kaiser test). This procedure was repeated for coupling the third amino acid and fatty acid, each of which was also added at 4 equiv.

The cleavage of the fatty acid–peptide conjugate from resin was conducted by adding a mixture of TFA:H_2_O:TIPS (5.0 mL; 95:2.5:2.5 *v*/*v*) to the resin, and the mixture was stirred at room temperature for 2 h. The solution containing TFA was filtered from the column, and the filtrate was evaporated on a rotary evaporator to remove excess TFA. Di-isopropyl ether was added to the resulting crude product to yield a white solid precipitate. The ether was removed by decantation, and the precipitated compound was washed three times with diethyl ether. The resulting compound was dried in a rotary evaporator, purified by lyophilization for 24 h in a freeze-dryer (Thermo Savant, Holbrook, NY, USA), and validated using LC-MS/MS.

#### 2.2.2. General Procedure for Synthesizing C-Terminus Acid Conjugates

The synthesis of C-terminus carboxylic acid derivatives was similar to the procedure described for synthesizing the C-terminus amide, except for the resin choice and the first coupling step, as described below. The Wang resin was placed in a Bio-Rad column and swelled in DCM (2.0 mL) for 30 min, and the solvent was pushed out with positive pressure. The first amino acid (Fmoc-Asp(tBu)-OH) was placed in a scintillation vial; dissolved in DMF (2.0 mL); and DIC (100 mg), HOBt (54 mg), and DMAP (5 mg) were added to the vial. This mixture was sonicated for dissolution, added to the resin, and shaken on a vortex mixer for 6 h. The solvent was removed from the column, and the column was washed with DMF (3 × 2 mL) and DCM (3 × 2 mL) (confirmed by a negative Kaiser test). We then followed the same protocol described in Section 2.2.1.

#### 2.2.3. Procedures to Remove Trifluoracetic Acid Counterions from Fatty Acid–Peptide Conjugates by Counteranion Exchange

The trifluoroacetic acid counteranion was replaced with HCl by dissolving the white precipitate obtained after ether trituration in 5.0 mL of 0.1 M HCl solution, stirring for 15 min, and then adding 5 mL of acetonitrile. The soluble mixture was then dried in a dry ice bath and lyophilized overnight to yield a dry solid powder.

#### 2.2.4. Determination of the Molecular Structures and Quantities of Compounds

The reagents and the fatty acid–amino acid/peptide conjugates were analyzed using an LC-MS/MS system consisting of an Agilent 1100 LC system (HPLC-DAD-autosampler, Agilent Technologies, Santa Clara, CA, USA) and a QTRAP 6500^+^ quadruple-linear trap mass spectrophotometer with electrospray ionization (AB Sciex LLC, Framingham, MA, USA). The nuclear magnetic resonance (NMR) analysis was conducted using a Bruker 400 MHz NMR instrument, DMSO-d_6_ (CAS No. 2206-27-1, 99.9 atom% D, Thermo-scientific, Fair Lawn, NJ, USA) as a solvent, and Topspin 4.3.0 version software. The ^1^H NMR (400 MHz), ^13^C NMR (100 MHz), and ^13^C DEPT data were acquired using a purified compound (30 mg) dissolved in 0.7 mL of DMSO-d_6_ in a 5 mm diameter NMR tube. DEPT 135 was used to determine the multiplicity of carbon atoms, and CH_2_ groups showed inverted signals, whereas CH and CH_3_ groups were upright. The quaternary carbon (C) did not show any signal.

### 2.3. Hydrogel Formation

#### 2.3.1. Hydrogel Preparation

Lyophilized fatty acid–peptide conjugates were dissolved in phosphate-buffered saline (PBS) at a final concentration of 0.5% (5.0 mg in 1 mL of PBS), 1.5% (15.0 mg in 1 mL of PBS), and 3% (30.0 mg in 1 mL of PBS). The pH of the peptide solutions was increased to 9.0 by adding 0.1 M NaOH to dissolve the compounds and readjusted to pH 2–8 by the drop-wise addition of 0.1 N HCl, followed by sonication. Most compounds formed hydrogels immediately after sonication; some were maintained overnight at room temperature for gel formation. The gel formation was confirmed by the vial inversion method, and photographs were taken.

#### 2.3.2. Hydrogel Sterilization

PBS, 0.1 N HCl, 0.1 M NaOH, pipette tips, and Eppendorf tubes were autoclaved (Steris, AMSCO 250LS, Conroe, TX, USA) at 130 °C for 45 min. The other procedures for hydrogel formation were performed under pathogen-free conditions inside a BSL-2 hood.

### 2.4. Rheological Studies

Tests were performed on 50 µL hydrogel samples using an Anton Paar MCR 092 rheometer (Anton Paar USA, Houston, TX, USA) with a 20 mm cone plate at a measuring gap of 39 µm. The effects of concentration on gel strength and viscoelastic behavior were assessed by conducting amplitude/strain sweep experiments for all gels using oscillatory shearing strain. The storage (G′) and loss (G″) moduli were measured as a function of strain (ranging from 0.01 to 100%) at a constant frequency of 10 rad/s. The mechanical stability of the peptide hydrogels was tested by running frequency sweep experiments at angular frequencies ranging from 1 to 100 rad/s at a constant 1% strain, which was under the limit of the linear viscoelastic region obtained from the amplitude sweep test. The hydrogel structure survived the tests under this strain. A time sweep curing experiment was conducted with a constant strain 10% and frequency 1 Hz. We studied the thixotropic properties to understand the time-dependent shear thinning of gels under 4 min of constant high shear strain (200%) that liquefied the gel. We then followed the re-gelation after the shear strain returned to the low shear strain of 0.1% used for the initial 2 min test. The G′ and G″ values represent the elastic gel-like and viscous liquid behaviors of our samples, respectively [41].

### 2.5. Hydrogel Swelling Ratio (%)

The swelling ratio assay was used to determine the water intake capacity of the hydrogels. First, different gel concentrations (1.5% and 3.0% *w*/*v*) were formed in PBS in preweighed vials (250 μL of gel in each vial). Next, 2 mL of PBS was added to the vials, and the gels were left to swell at different times. The excess PBS was then removed from the vial, and the vials were weighed. The swelling ratio (%) of the hydrogel was calculated using the following equation:

Swelling ratio (%) = [(Wt − Wi)/Wi] × 100

where Wt is the weight of the swollen hydrogel at a specific time point t, and Wi is the initial weight of the hydrogel.

### 2.6. Field Emission Scanning Electron Microscopy

The 3% *w*/*v* hydrogel was freeze-dried at −80 °C and then lyophilized under vacuum to obtain a fine powder. Field emission scanning electron microscopy (FESEM) analysis of the lyophilized hydrogel was conducted on an S4800 field emission scanning electron microscope (Hitachi, Santa Clara, CA, USA) under a high vacuum to assess the surface structure of the freeze-dried peptide-based hydrogel according to published and widely used procedures [42,43]. The following parameters were used: stage distance, 12 mm; acceleration voltage, −3.0 kV; and working distance, 2.2 mm. The sample was initially sputter coated with a thin carbon layer for increased conductivity. A high magnification allowed for the observation of the fibrous network on the top layer of the hydrogel samples.

### 2.7. Hydrogel Treatment of Third-Degree Burn Wounds Generated on Mice and Histological Study of Treated Wounds

The animal use protocol was authorized and approved by the Institutional Animal Care and Use Committee of Louisiana State University Health Sciences Center, New Orleans, and followed the ARRIVE guidelines [44]. Briefly, full-thickness burn wounds (6 mm diameter) were generated in the dorsal skin on both sides along the midline of *C57BL/6J* mice (female, 18 months old, Jackson Laboratory, Bar Harbor, ME, USA) at 0 days post burn (dpb), similar to the procedures performed by us and others previously [1,45,46,47,48,49]. Each treatment group included four mice. We excised a 6 mm diameter circle of coagulated or necrotic full-thickness skin at the center of each burn wound at 48 h (2 dpb) and filled each excision-generated space with hydrogel (30 µL) by injection. The wounds were covered with Tegaderm waterproof dressings to protect the tissue and hydrogel in the wounds and to prevent water loss. Each wound was covered with two pieces of a 50.8 mm long and 30 mm wide Tegaderm waterproof film dressing (cut from a roll of the product 50.8 mm wide × 10,058 mm long, catalog number 16002, 3M, Saint Paul, MN, USA) to protect the tissue and hydrogel in the wounds and to prevent water loss, one was on the dorsal side, and the other was on the abdominal side. The two film pieces were placed end to end and overlapped each other, forming a cylinder-shaped adhesive belt wrapping around the skin of mouse body trunk. This film belt adhered to the skin, including the wound margins, from the dorsal to abdominal side, all around the mouse body trunk. This method of film application allows the film to reduce skin contraction, facilitating wound closure through re-epithelialization, as described in prior studies [50,51,52], thus better resembling wound healing in humans. We used swabs to clean the area of skin surface to allow the film to stick to the skin surface tightly. The adhesion of a film end to the end of another film is much stronger than adhesion of film to the skin. In turn, our method of wrapping wounds with Tegaderm film allowed the film to adhere to skin around wounds for the intended duration even when the mice were active, which is likely to be more effective and durable than those in references [50,51,52] for wound contract control using Tegaderm film. The film was changed at 2 dpb for the excision/debridement of the necrotic tissue of the burn wound. To clearly photograph the wound area, we also changed the film and hydrogel at Day 7 post burn. As the renewal only took a few minutes, it should not affect the control of wound contraction. The excision mimics the practice of debriding severely burned skin. The same type of wounds without hydrogel treatment were used as the control. The mice were anesthetized with ketamine and xylazine (100 and 10 mg/kg, respectively, i.p.) prior to any wounding. Sustained-release buprenorphine was also injected (s.c., 1 mg/kg) for analgesia. We monitored mouse health and behavior, including drinking, foraging, grooming, and eating, and we examined all wounds twice per day to ensure that the hydrogel and dressing stayed in place. Wound healing was assessed as described previously [53,54,55,56,57,58]. The wounds were photographed, and their areas were calculated using NIH ImageJ software version 1.54d. The wound closure was reported as the percentage of the closed wound area compared with the initial burn wound area. The mice were euthanized, and burn wounds with 3 mm skin rims were excised and histologically studied after hematoxylin–eosin staining, as we did previously [59,60]. The livers, kidneys, and spleens were also collected, weighed, and measured for size to obtain a gross assessment of toxicity. The sections stained with hematoxylin–eosin were photographed using an OLYMPUS scanning microscope (OLYMPUS, Tokyo, Japan). The epithelial gap—i.e., the distance between the neoepithelium emerging from the edges of the wound area—was measured using OLYMPUS OlyVIA software version 3.4.1 (Build 26606), as described previously but with some modifications [53,60,61].

### 2.8. Statistical Analysis

Statistical analysis was conducted using *t* tests or ANOVA via GraphPad Prism 9.0 software, and *p* < 0.05 was considered statistically significant. Data are presented as mean ± standard error of mean (SEM) or standard derivation (SD).

## 3. Results and Discussion

### 3.1. Design, Synthesis, and Hydrogelability Tests of Novel Covalent Fatty Acid–Tripeptide Conjugates

Our aim was to develop amphiphilic conjugates as innovative hydrogelators, with each hydrogel containing a fatty acid covalently bonded to a peptide and capable of self-assembling into a hydrogel with the hydrophobic tail from the linear carbon chain of fatty acid and the hydrophilic head from the peptide domain. To this effect, we designed and synthesized a large panel of compounds through SPPS, including peptides inspired by the pentapeptide KYFIL [36]. The amphiphilic conjugates of these peptides and fatty acids were also synthesized. Unfortunately, these compounds were unable to form hydrogels. Based on our findings from these unreported trials and the inspiration from the LIVAGD hydrogelator template [38], we focused on synthesizing a fatty acid-conjugated tripeptide possessing a molecular template of a fatty acid (with n carbons) coupled to an amino acid1–amino acid2–aspartic acid tripeptide (Cn acid–AA1–AA2–D). This coupling generates novel amphiphilic molecules that can self-assemble into hydrogels within the living pathophysiological niche. AA1 and AA2 are hydrophobic amino acids such as alanine (A), valine (V), isoleucine (I), or leucine (L) that play a role in self-assembly. Aspartic acid (D), an amino acid with a hydrophilic carboxylic acid side chain, forms the hydrophilic head at the tripeptide terminus and offers polarity and aqueous solubility. The C-terminus polar hydrophilic end (carboxyl or amidated carboxyl) then improves solubility in aqueous media, while the side chain carboxylic acid group further increases the hydrophilic character of the hydrogel in solution. The fatty acid, either octanoic (C8) acid, dodecanoic/lauric (C12) acid, tetradecanoic/myristic (C14) acid, or hexadecanoic/palmitic (C16) acid, was predicted to participate in self-assembly due to its hydrophobicity and the shape of the long carbon chain. Shorter fatty acids (such as C6 acid) are likely not to be hydrophobic enough for hydrogelation.

#### 3.1.1. Design, Synthesis, and Hydrogelability Tests of Cn Fatty Acid-ILD-NH_2_-Type Conjugates

**Figure 1:**
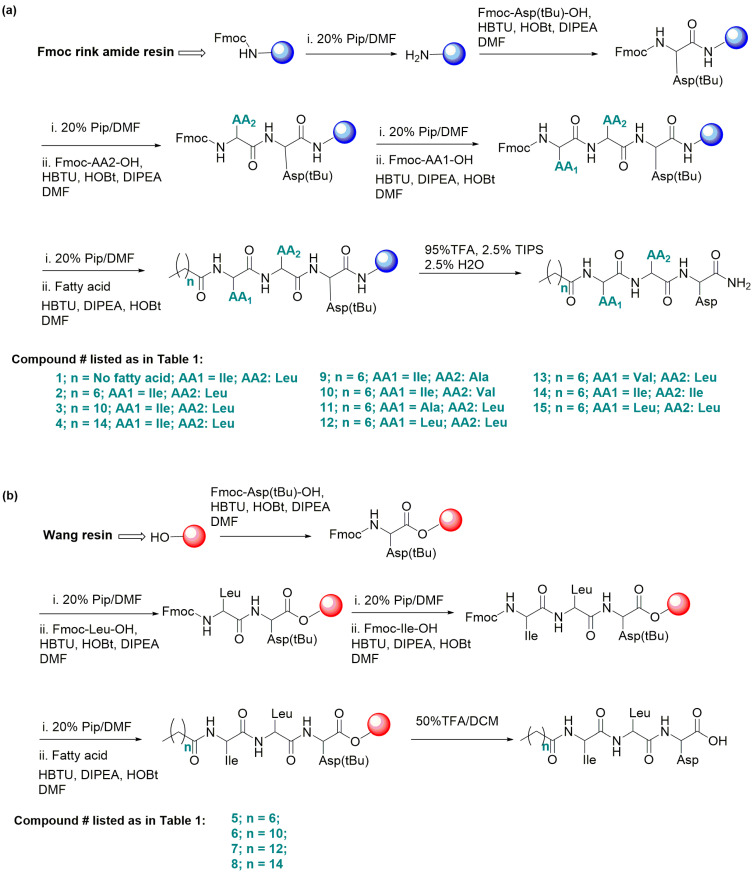
General Fmoc/tBu-based solid-phase peptide synthesis (SPPS). (**a**) For peptides with a C-terminal amide (–NH_2_), Rink amide resin was used (for compounds **1**–**4** and **9**–**15**). (**b**) For peptides with a C-terminal acid (-OH), Wang resin was used (for compounds **5**–**8**).

We tested this design by initially synthesizing compound (**1**) (H-ILD-NH_2_) with the C-terminal carboxyl bonded to an amide (–NH_2_) but with the N-terminal intact (H) using Rink-amide-resin-based SPPS (Figure 1a, Table 1) to find if the tripeptide itself undergoes gelation. We observed that it did not gelate in PBS.

**Figure 2:**
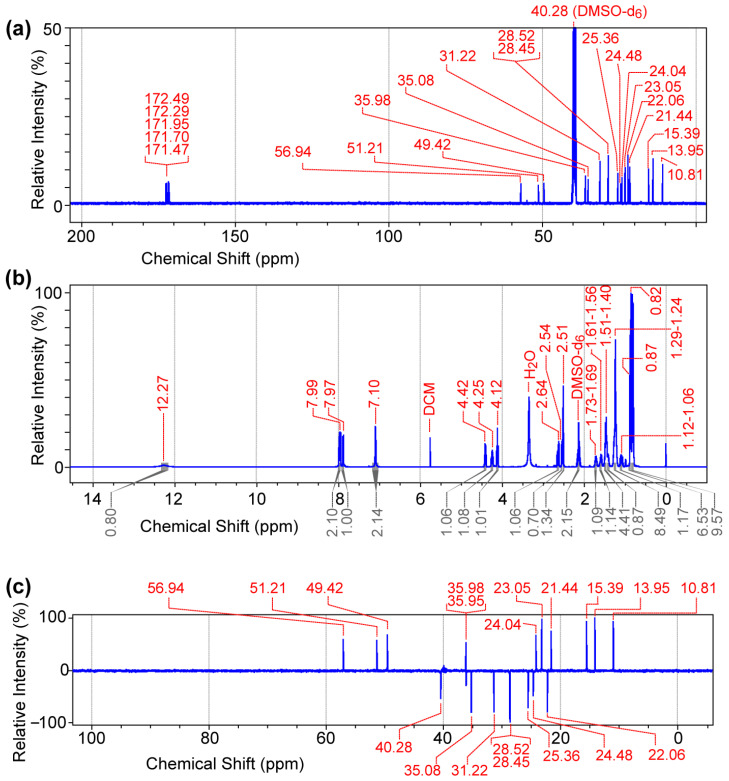
NMR spectra of caprylic acid–isoleucine–leucine–aspartic acid amide conjugate [C8 acid-ILD-NH_2_(**2**)]. (**a**) ^13^C NMR (100 MHz). (**b**) ^1^H NMR (400 MHz). (**c**) ^13^C DEPT-135 NMR (100 MHz). Data were acquired for purified (**2**) (30 mg) dissolved in 0.7 mL of DMSO-d_6_.

We then synthesized compound **2** [C8-ILD-NH_2_(**2**)] with a C8 fatty acid linked to the N-terminus of (**1**) (tripeptide ILD–NH_2_) via an amide bond (Figure 1a). The molecular structure and purity of the synthesized C8-ILD-NH_2_(**2**) were determined using NMR and LC-MS/MS. The NMR spectra illustrated in Figure 2 indicate chemical shifts δ and coupling constants *J* that clearly verify the structure of (**2**) as the following: ^13^C NMR (100 MHz, DMSO-d_6_) (Figure 2a): δ 172.49, 172.29, 171.95, 171.70, 171.47, 56.94, 51.21, 49.42, 40.28 (DMSO-d_6_), 35.98, 35.95, 35.08, 31.22, 28.52, 28.45, 25.36, 24.48, 24.04, 23.05, 22.06, 21.44, 15.39, 13.95, 10.81; ^1^H NMR (400 MHz, DMSO-d_6_) (Figure 2b): δ 12.27 (brs, 1H), 7.99 (d, *J =* 8.4 Hz, 1H), 7.97 (dd, *J* = 12.2, 8.4 Hz, 2H), 7.10 (d, *J* = 8.4 Hz, 2H), 4.42 (dd, *J* = 8.4, 6.8 Hz, 1H), 4.25 (dd, *J* = 8.4, 6.0 Hz, 1H), 4.12 (t, *J* = 6.0 Hz, 1H), 2.64 (dd, *J* = 8.4, 6.0 Hz, 1H), 2.54 (d, *J* = 7.2 Hz, 1H), 2.51 (dd, *J* = 8.4, 3.7 Hz, 1H), 2.17–2.10 (m, 2H), 1.73–1.69 (m, 1H), 1.61–1.56 (m, 1H), 1.51–1.40 (m, 5H), 1.29–1.24 (m, 8H), 1.12–1.06 (m, 1H), 0.87 (s, 6H), 0.82 (s, 9H); and ^13^C DEPT-135 NMR (100 MHz, DMSO-d_6_) (Figure 2c): 56.94 (C_α_H), 51.21 (C_α_H), 49.42 (C_α_H), 40.28 (CH_2_), 35.98 (CH), 35.95 (CH_2_), 35.08 (CH_2_), 31.22 (CH_2_), 28.52 (CH_2_), 28.45 (CH_2_), 25.36 (CH_2_), 24.48 (CH_2_), 24.04 (CH), 23.05 (CH_3_), 22.06 (CH_2_), 21.44 (CH_3_), 15.39 (CH_3_), 13.95 (CH_3_), 10.81 (CH_3_).

**Figure 3:**
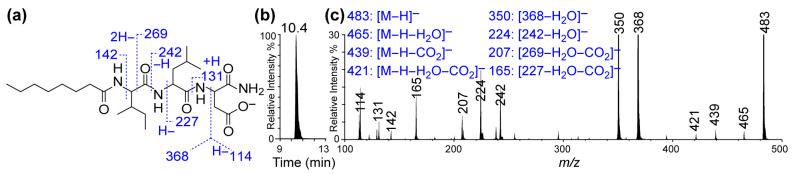
LC-MS/MS of caprylic acid–isoleucine–leucine–aspartic acid amide conjugate [C8 acid-ILD-NH_2_(**2**)]. (**a**) Deprotonated molecular structure of C8 acid-ILD–NH_2_(**2**) and interpretative illustration of its MS/MS fragment ions. (**b**) A typical C18 reversed-phase chromatogram from LC-MS/MS analysis of (**2**). (**c**) LC-MS/MS spectrum of (**2**) and interpretation of its MS/MS fragment ions (right). The LC-MS/MS system consisted of an Agilent 1100 LC and QTRAP 6500^+^ quadruple-linear trap mass spectrophotometer with electrospray ionization.

The LC-MS/MS spectrum confirmed a molecular mass M of 484 Daltons, while the fragment ions *m*/*z* (single charge, z = 1): 114, 131, 142, 165, 207, 224, 242, 350, 368, 421, 439, 465, and 483 [M − H^+^] offered fingerprints for the structure of C8-ILD-NH_2_(**2**) (Figure 3). The fragment ions corresponding to the details of the molecular structure are interpreted in Figure 3a. The LC-MS analysis also indicated that (**2**) was highly pure (>96%). When we dissolved C8-ILD-NH_2_(**2**) into PBS by raising the pH to pH 9 and then reducing it to pH 7.4, it formed hydrogels (Table 1), while it also formed hydrogels if PBS was replaced with deionized water. These results supported the suitability of our synthetic route and the procedures illustrated in Figure 1a for synthesizing Cn acid–AA1–AA2–D–NH_2_-type compounds while demonstrating their potential hydrogelability.

**Figure 4:**
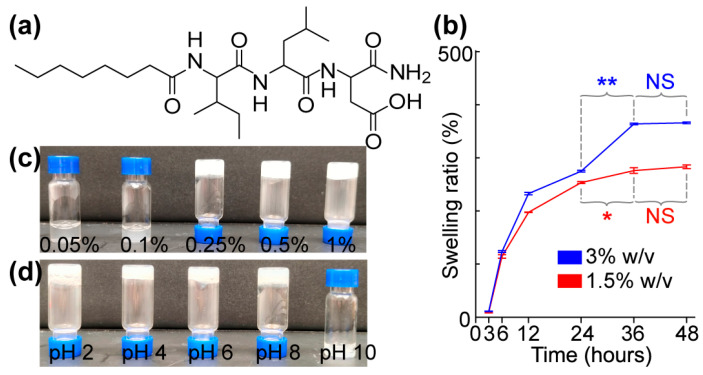
Molecular structure, gelable concentration, and pH-related stability of the hydrogel self-assembled from C8-ILD-NH_2_(**2**). (**a**) Molecular structure of (**2**). (**b**) Swelling ratio at 1.5% and 3% (*w*/*v*). (**c**) Gelation at different concentrations of (**2**) (*w*/*v*) in PBS at pH 7.4: 0.05%, no gelation; 0.1%, no gelation; 0.25%, gelated; 0.5%, gelated; 1.0%, gelated. (**d**) Photos showing the gel stability of C8-ILD-NH_2_(**2**) (1.5% *w*/*v*) at different pHs: pH 2–8, gelated and stable; pH 10, no gelation. Results are presented as mean ± SD (*n* = 3) with * *p* < 0.05, ** *p* < 0.01. NS: no significant difference.

Compounds **3** [C12-ILD-NH_2_)] and **4** [C16-ILD-NH_2_], with C12 and C16 fatty acids, respectively, linked to the N-terminus of (**1**) (tripeptide ILD–NH_2_) by an amide bond, were then synthesized, as illustrated in Figure 1a. We determined the hydrogelability of both compounds following the same protocol used for (**2**) after the verification of their structures and purity (>95%) through LC-MS/MS. Both (**3**) and (**4**) formed hydrogels similar to (**2**) in PBS buffer at ≥ 0.25% (*w*/*v*) concentration and remained as a solution below 0.25% (*w*/*v*) (Figure 4c). The key difference in the gelation observed between (**2**), (**3**), and (**4**) was that (**2**) instantly formed a hydrogel upon sonication, whereas (**3**) and (**4**) took more time under the same conditions. Moreover, when shaken by hand, hydrogel (**2**) was more stable than either (**3**) or (**4**), suggesting a more compact packing of shorter C8 carboxylic acids. The selected compound C8-ILD-NH_2_(**2**) gelates in 2 min after the pH was adjusted to 7.4 (Video S1), whereas most of the other compounds in Table 1 form gels immediately within 2–15 min after the pH was adjusted to 7.4. The gelation time increased as the carbon of Cn fatty acids increased from 8 to 16 carbons.

#### 3.1.2. Design, Synthesis, and Hydrogelability Tests of Cn Fatty Acid–ILD–OH–Type Conjugates

The C-terminal carboxyl of compounds (**2**), (**3**), and (**4**) is amidated with a primary –NH_2_ group. We questioned whether the hydrogelability would change if this carboxyl was not amidated, and we addressed this possibility by synthesizing compounds **5** [C8–ILD–OH(**5**)], **6** [C12–ILD–OH (**6**)], **7** [C14–ILD–OH (**7**)], and **8** [C16–ILD–OH (**8**)] at high purity (>95%) via Wang-resin-based SPPS (Figure 1b) with C8, C12, C14, and C16 fatty acids, respectively, bonded to the N-terminus of ILD-OH. The representative NMR and LC-MS/MS data, which confirmed the molecular structures of these compounds, are as follows:

C12–ILD–OH (**6**). ^1^H NMR (in DMSO-d_6_) (Figure S1): δ 12.5 (brs, 2H), 8.06 (d, *J* = 8.4 Hz, 1H), 7.91 (d, *J* = 8.4 Hz, 1H), 7.83 (d, *J* = 8.4 Hz, 1H), 4.50 (dd, *J* = 7.5, 6.6 Hz, 1H), 4.36 (dd, *J* = 8.4, 7.0 Hz, 1H), 4.16 (t, *J* = 7.5 Hz, 1H), 2.68–2.55 (m, 2H), 2.53–2.51 (m, 1H), 2.16–2.07 (m, 2H), 1.72–1.69 (m, 1H), 1.62–1.56 (m, 1H), 1.48–1.42 (m, 4H), 1.29–1.24 (brs, 16H), 1.11–1.04 (m, 1H), 0.87 (t, *J =* 7.5 Hz, 6H), and 0.82 (t, *J* = 7.5 Hz, 9H); ^13^C NMR (100 MHz) and ^13^C DEPT NMR, DMSO-d_6_ (Figures S2 and S3): δ 172.2, 172.1, 171.6, 171.6, 171.6, 171.0, 56.6, 50.6, 48.4, 40.8, 36.1, 35.8, 35.1, 31.3, 29.0, 28.9, 28.8, 28.7, 28.5, 25.4, 24.3, 24.0, 23.0, 22.1, 21.4, 15.3, 13.9, and 10.7. MS/MS fragmentation ions verified the molecular mass and structure (Table 1).

C14–ILD–OH (**7**). ^1^H NMR ((in DMSO-d_6_) (Figure S4): δ 12.51 (brs, 2H), 8.07 (d, *J* = 9.0 Hz, 1H), 7.91 (d, *J* = 9.0 Hz, 1H), 7.83 (d, *J* = 9.0 Hz, 1H), 4.52 (q, *J* = 7.6 Hz, 1H), 4.34 (dd, *J* = 7.6 Hz, 1H), 4.16 (t, *J* = 7.6 Hz, 1H), 2.68–2.52 (m, 2H), 2.53–2.51 (m, 1H), 2.16–2.09 (m, 2H), 1.75–1.69 (m, 1H), 1.62–1.55 (m, 1H), 1.47–1.43 (m, 4H), 1.25 (brs, 20 H), 1.11–1.04 (m, 1H), 0.87 (t, *J* = 7.5 Hz, 6H), and 0.82 (t, *J* = 7.5 Hz, 9H); ^13^C NMR (100 MHz) and ^13^C DEPT NMR, DMSO-d_6_ (Figures S5 and S6): δ 172.2, 172.1, 171.6, 171.6, 171.6, 171.0, 56.6, 50.6, 48.4, 40.8, 36.1, 35.8, 35.1, 31.3, 29.0, 29.0, 28.9, 28.8, 28.7, 28.5, 25.4, 24.3, 24.0, 23.0, 22.1, 21.4, 15.3, 13.9, and 10.7. MS/MS fragmentation ions confirmed a molecular mass and structure (Table 1).

C16–ILD–OH (**8**). ^1^H NMR (in DMSO-d_6_) (Figure S7): δ 12.50 (brs, 2H), 8.06 (d, *J* = 8.0 Hz, 1H), 7.91 (d, *J* = 8.0 Hz, 1H), 7.83 (d, *J* = 8.6 Hz, 1H), 4.52 (q, *J* = 7.6 Hz, 1H), 4.34 (dd, *J* = 7.6 Hz, 1H), 4.16 (t, *J* = 7.6 Hz, 1H), 2.68–2.55 (m, 2H), 2.53–2.51 (m, 1H), 2.17–2.07 (m, 2H), 1.74–1.68 (m, 1H), 1.61–1.56 (m, 1H), 1.48–1.43 (m, 4H), 1.24 (brs, 28 H), 1.11–1.04 (m, 1H), 0.87 (t, *J* = 7.5 Hz, 6H), and 0.82 (t, *J* = 7.5 Hz, 9H); ^13^C NMR (100 MHz) and ^13^C DEPT NMR, DMSO-d_6_ (Figures S8 and S9): δ 172.2, 172.1, 171.6, 171.6, 171.0, 56.6, 50.6, 48.4, 40.9, 36.1, 35.9, 35.1, 31.3, 29.0, 29.0, 28.9, 28.8, 28.7, 28.5, 25.4, 24.3, 24.0, 23.0, 22.1, 21.4, 15.4, 13.9, and 10.7. MS/MS fragmentation ions verified the molecular mass and structure (Table 1).

C8–ILD–OH (**5**), the C-terminal carboxylic acid counterpart of C8-ILD-NH_2_(**2**)**,** was unable to form a hydrogel at all, unlike (**2**) (Table 1). Hydrogels were formed from the other tested Cn acid–ILD–OHs (C12–ILD–OH (**6**), C14–ILD–OH (**7**), and C16–ILD–OH (**8**) with C12, C14, and C16 fatty acids, respectively, bonded to the N-terminus of ILD-OH). However, these hydrogels were weaker than those formed by Cn acid-ILD-NH_2_ with the same Cn acid when shaken by hand. Thus, the primary amide offers better gelation than its carboxylic acid counterpart when attached to the C-terminus of the Cn acid-ILD molecular framework. Of note, MS/MS fragmentation ions verified the molecular masses and were structured as (**5**), (**6**), (**7**), and (**8**) (Table 1).

#### 3.1.3. Design, Synthesis, and Hydrogelability of More Novel C8 Fatty Acid–AA1–AA2–D–NH_2_-Type Conjugates

We also synthesized different analogs of C8-ILD-NH_2_(**2**), in which the first 2 amino acids I (AA_1_) and L (AA_2_) were swapped with each other or replaced by other hydrophobic amino acids (e.g., alanine (A) and valine (V)). The same Rink-amide-resin-based SPPS outlined in Figure 1a for the organic synthesis of (**2**), was used for the generation of these novel analogs. The representative NMR and LC-MS/MS data that verified the molecular structures and purities (>95%) of these analogs are as follows:

C8–IAD–NH_2_(**9**). ^1^H NMR (400 MHz, DMSO-d_6_) (Figure S10): δ 12.18 (brs, 1H), 8.85 (d, *J* = 9.8 Hz, 1H), 8.38 (t, *J* = 7.7 Hz, 1H), 8.08 (d, *J* = 6.8 Hz, 1H), 7.88 (t, *J* = 7.6 Hz, 2H), 7.12 (d, *J* = 5.3 Hz, 2H), 4.42 (q, *J* = 7.2 Hz, 1H), 4.24–4.16 (m, 1H), 4.13 (t, *J* = 7.95 Hz, 1H), 2.63 (dd, *J* = 7.2, 6.0 Hz, 1H), 2.55 (d, *J* = 7.2 Hz, 1H), 2.20–2.08 (m, 2H), 1.74–1.69 (m, 1H), 1.53–1.40 (m, 3H), 1.28–1.24 (m, 9H), 1.21 (d, *J* = 7.1 Hz, 4H), 1.14–1.05 (m, 1H), 0.86 (t, *J* = 6.9 Hz, 3H), and 0.82 (t, *J* = 6.9 Hz, 6H); ^13^C NMR (100 MHz) and ^13^C DEPT NMR, DMSO-d_6_ (Figures S11 and S12): δ 172.5, 172.3, 171.9, 171.3, 144.5, 143.1, 126.2, 56.8, 49.3, 48.5, 36.2, 35.9, 35.0, 31.2, 28.5, 28.4, 25.3, 24.4, 22.0, 17.6, 15.4, 13.9, and 10.9; MS/MS fragmentation ions verified the molecular mass and structure of (**9**) (Table 1).

C8-IVD-NH_2_(**10**). ^1^H NMR (400 MHz, DMSO-d_6_) (Figure S13): δ 12.29 (brs, 1H), 8.05 (d, *J* = 7.7 Hz, 1H), 7.90 (d, *J* = 7.6 Hz, 1H), 7.76 (d, *J* = 7.7 Hz, 1H), 7.10 (brs, 2H), 4.45 (d, *J* = 7.5 Hz, 1H), 4.18 (t, *J* = 8.1 Hz, 1H), 4.10 (t, *J* = 7.4 Hz, 1H), 2.64 (dd, *J* = 7.4, 5.7 Hz, 1H), 2.56–2.49 (m, 1H), 2.17–2.10 (m, 2H), 1.96–1.94 (m, 1H), 1.75–1.69 (m, 1H), 1.51–1.40 (m, 3H), 1.23 (brs, 8H), 1.12–1.06 (m, 1H), 0.87 (s, 6H), and 0.80 (s, 9H); ^13^C NMR (100 MHz) and ^13^C DEPT NMR, DMSO-d_6_ (Figures S14 and S15): δ 172.3, 172.1, 171.8, 171.5, 170.6, 57.8, 56.8, 49.3, 35.9, 35.8, 35.0, 31.2, 30.3, 28.5, 28.4, 25.4, 24.4, 22.0, 19.1, 18.0, 15.4, 13.9, and 10.7; MS/MS fragmentation ions verified the molecular mass and structure of (**10**) (Table 1).

C8-ALD-NH_2_(**11**). ^1^H NMR (400 MHz, DMSO-d_6_) (Figure S16): δ 12.27 (brs, 1H), 7.97 (dd, *J* = 12.2, 8.4 Hz, 2H), 7.99 (d, *J* = 8.4 Hz, 1H), 7.10 (d, *J* = 8.4 Hz, 2H), 4.42 (dd, *J* = 8.4, 6.8 Hz, 1H), 4.25 (dd, *J* = 8.4, 6.0 Hz, 1H), 4.12 (t, *J* = 6.0 Hz, 1H), 2.64 (dd, *J* = 8.4, 6.0 Hz, 1H), 2.54 (d, *J* = 7.2 Hz, 1H), 2.51 (dd, *J* = 8.4, 3.7 Hz, 1H), 2.17–2.10 (m, 2H), 1.73–1.69 (m, 1H), 1.61–1.56 (m, 1H), 1.51–1.40 (m, 5H), 1.29–1.24 (m, 8H), 1.12–1.06 (m, 1H), 0.87 (s, 6H), and 0.82 (s, 9H); ^13^C NMR (100 MHz) and ^13^C DEPT NMR, DMSO-d_6_ (Figures S17 and S18): δ 172.8, 172.4, 172.3, 171.9, 171.7, 51.4, 49.3, 48.2, 40.3, 35.8, 35.0, 31.1, 28.5, 28.4, 25.1, 24.0, 23.0, 22.0, 21.5, 17.6, and 13.9. MS/MS fragmentation ions verified the molecular mass and structure of (**11**) (Table 1).

C8-LLD-NH_2_(**12**). ^1^H NMR (400 MHz, DMSO-d_6_) (Figure S19): δ 8.09–8.02 (m, 2H), 7.54 (brs, 1H), 7.00 (brs, 2H), 4.36–4.28 (m, 2H), 4.18 (q, *J* = 7.1 Hz, 1H), 4.04 (q, *J* = 7.1 Hz, 1H), 2.41–2.39 (m, 1H), 2.13–2.09 (m, 1H), 1.64–1.56 (m, 2H), 1.49–1.45 (m, 4H), 1.24 (brs, 8H), 1.12–1.06 (m, 1H), and 0.89–0.86 (m, 12H); ^13^C NMR (100 MHz) and ^13^C DEPT NMR, DMSO-d_6_ (Figures S20 and S21): δ 172.8, 172.4, 172.3, 171.9, 171.7, 51.4, 49.3, 48.2, 40.3, 35.8, 35.0, 31.1, 28.5, 28.4, 25.1, 24.0, 23.0, 22.0, 21.5, 17.6. The MS/MS fragmentation ions verified the molecular mass and structure of (**12**) (Table 1).

C8-VLD-NH_2_(**13**), C8-IID-NH_2_(**14**), and C8-LID-NH_2_(**15**) structures were verified using the same NMR and LC-MS/MS methods as for the other Cn acid–AA1–AA2–D compounds above (Figures S22–S27, Table 1).

The Cn acid–AA1–AA2–D-NH_2_ compounds (**9**–**15**) can all self-reassemble into hydrogels in PBS at a 1.5% *w*/*v* concentration when subjected to gelation conditions. All compounds formed hydrogels (Table 1).

### 3.2. Determination of Wound Compatibility, Injectability, and Rheological Properties of a Novel Hydrogel Formed from a Selected Cn Fatty Acid–AA1–AA2–D Conjugate

We explored the suitability of our novel hydrogels for accelerating the healing of third-degree burn wounds by exploring the biocompatibility, toxicity, and biodegradability of C8-ILD-NH_2_(**2**) as a representative compound chosen from the 13 hydrogelating Cn acid–AA1–AA2–D type-conjugates compiled in Table 1. We chose (**2**) because it showed more rapid gelation and formed a more stable hydrogel in saline compared with the other conjugates, based on our visual observations (Table 1, Figure 4). Swelling in aqueous environments is an important characteristic of hydrogels and is mostly determined using the pore size, the intermolecular forces inside the hydrogel network structure, and the hydrophilic nature of the hydrogel [62]. Hydrogels with large pore sizes and sufficient hydrophilicity can take up large amounts of water (liquid/fluid) and swell to weights multiple times greater than their own weight, and hydrogels with these qualities have been successfully applied for full-thickness skin wound healing and tissue regeneration [62,63] The strong swelling capacity is useful for absorbing leaked blood, exudate, and body fluids, and for transferring nutrients and metabolites. The swelling ratios of hydrogel (**2**) at concentrations of 1.5% (red) and 3% (blue) (*w*/*v*) were 197.3 and 232.6%, respectively, after 12 h of PBS absorption at a physiological pH of 7.4 (Figure 4b). These percentages continuously increased to 276.0% and 364.0%, respectively, at 36 h. However, the swelling behavior subsequently reached a plateau of 276.0–283.3% and 364.0–366.0%, respectively, from 36 h to 48 h. Hydrogel (**2**) would clearly be able to take up unwanted discharge/exudate from wounds to promote wound healing.

We determined the gelability of C8-ILD-NH_2_(**2**) at concentrations from 0.05% to 1% (*w*/*v*) using a vial inversion test (Figure 4c). C8-ILD-NH_2_(**2**) gelated in PBS at concentrations ≥0.25% (*w*/*v*) but remained a solution at lower concentrations. We also tested the C8-ILD-NH_2_(**2**) gel from pH 2 to pH 10 to reflect the pH changes in human wounds under various treatments [64,65]. C8-ILD-NH_2_(**2**) formed a hydrogel and stayed gelated from pH 2 to pH 8 (Figure 4d), confirming its ability to form a stable hydrogel in PBS at a concentration as low as 0.25% and within a pathophysiologically relevant pH range.

The injectability of a hydrogel determines its capability to precisely fill 3D surfaces and cavities as a liquid and then solidify onsite as an elastic matrix. The matrix can be engineered to comply with mechanical and metabolic needs, including the delivery of drugs or stem cells for the recovery of locally damaged cells and tissues. For these purposes, a hydrogel will be more versatile and have minimal potential to induce itching or pain in wound treatment if it is injectable and compliant with the internal strain from cell/tissue regeneration or remodeling, as well as any external strains that the wound can experience, such as compression or shearing between the wound surface and wound dressing. Gelation time is a very important parameter for injectability, which was used to further evaluate our hydrogels (Table 1). C8-ILD-NH_2_(**2**) gelated in 2 min after the pH was adjusted to 7.4 as shown in Video S1 in the Supplemental Materials, while other compounds in Table 1 gelated within 2–15 min after the pH was adjusted to 7.4. Gelation times for Cn-ILD-NH_2_-type compounds increased from 2 to 10 min when the n of Cn fatty acid increased from 8 to 16 carbons. Gelation times for Cn-ILD-OH-type compounds increased from 7 to 15 min when the n of Cn fatty acid increased from 12 to 16 carbons, but when the n reduced from 12 to 8 (i.e., from Cn-ILD-OH (**6**) to C8-ILD-OH (**5**)), the gelability lost (Table 1). These results indicate that n needs to be more than 8 for Cn-ILD-OH-type compounds to gelate. With the increase in the carbon chain length Cn, there was an increase in the hydrophobicity in the molecule. The increased hydrophobicity resulted in poor water retention and took more time for gelation. Gelation times were longer for C12-ILD-OH(**6**) (7 min) and C16-ILD-OH (**8**) (15 min), respectively, than for C12-ILD-NH_2_(**3**) (6 min) and C16-ILD-NH_2_(**4**) (10 min) when Cn acid is the same (Table 1). For C8-AA1–AA2–D-NH_2_-type compounds, the gelation times were in an increased order from left to right as follows: C8-ILD-NH_2_(**2**) (2 min) = C8-IAD-NH_2_(**9**) (2 min) < C8-IVD-NH_2_(**10**) (3 min) = C8-ALD-NH_2_(**11**) (3 min) < C8-LLD-NH_2_(**12**) (5 min) = C8-IID-NH_2_(**14**) (5 min) = C8-LID-NH_2_(**15**) (5 min) < C8-VLD-NH_2_(**13**) (6 min) (Table 1).

**Figure 5:**
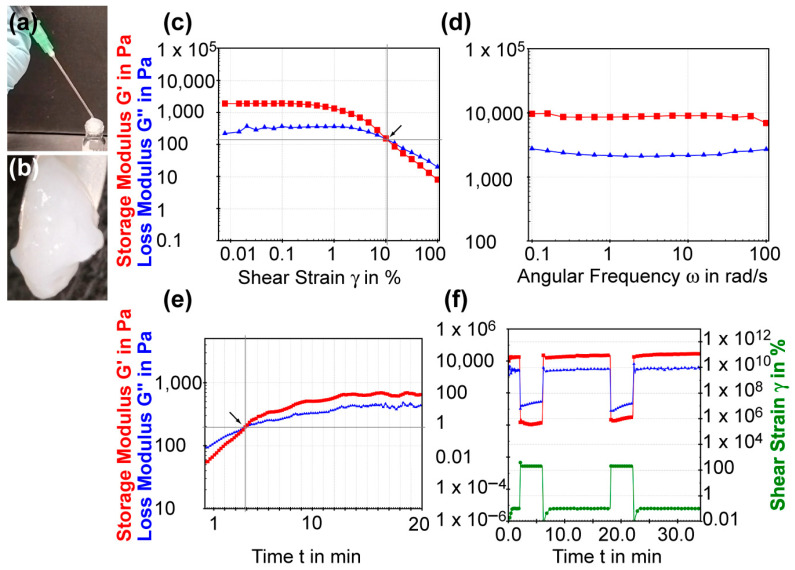
The injectable and rheological properties of C8-ILD-NH_2_(**2**) hydrogel. (**a**) Photo showing the injectable nature: injection by syringe. The hydrogel re-gelated quickly along the vial neck after injection. (**b**) A zoom-in photo of the re-gelated hydrogel after injection. (**c**) Shear strain amplitude sweep experiment of (**2**) with constant frequency of 10 rads/s. Arrow marks the flow point where G′ and G″ cross over. (**d**) Frequency sweep experiment with a constant shear strain of 1%. (**e**) Time sweep curing experiment with a constant strain (10%) and frequency (1 Hz). Arrow marks the crossover point of G′ and G″. (**f**) Thixotropic test with imposition of a gel-endurable shear strain of 0.1% and a gel-breaking shear strain of 200%. The rheological tests were conducted using (**2**) at 3.0% (*w*/*v*) in PBS at pH 7.4 and 37 °C.

We determined the injectability of the C8-ILD-NH_2_(**2**) hydrogel by tapping the vial rapidly and repeatedly on a table surface until the hydrogel liquefied. We then drew the liquid into a 1 mL syringe through a 25-gauge needle and quickly injected the liquid into the target site. We observed spontaneous re-gelation, as shown in the photos (Figure 5a,b) of this process.

The amplitude sweep test of the C8-ILD-NH_2_(**2**) hydrogel showed that it had a larger storage modulus G′ than loss modulus G″ in the limit of the linear viscoelastic region (on the left of the chart in Figure 5c), indicating the presence of an elastic gel structure. When the shear strain increase just surpassed 10.56%, the G′ and G″ curves crossed over each other (crossover point G′ = G″), so that G′ < G″, indicating a transition from the gel state to a viscous liquid state. Thus, the shear strain that promotes gel flow (i.e., the flow point) is 10.56%. The frequency sweep test (0.1–100 rad/s) of the C8-ILD-NH_2_(**2**) hydrogel at the 1% strain showed G′ (~10,000 Pa) > G″ (2–3 kPa), indicating a gel status under this condition (Figure 4d). It also showed that G″ and G′ readouts were independent of the frequency, reflecting the high stability of hydrogel (**2**). Hydrogels formed from 1%, 1.5%, and 3% C8-ILD-NH_2_(**2**) in PBS in our pilot study (Table 1, Figure 4) showed elastic moduli comparable to those of the dermis or skin (0.5–18 kPa) [39,40], supporting the suitability of our hydrogels for wound treatment. The isothermal time-sweep curing test for gel formation (Figure 5e) from the G′ < G″ sol state to G′ > G″ gel state showcased the gel formation of (**2**) and a crossover point at 3.87 min (G′ = G″) under a constant 10% shear strain (amplitude) and constant 1 Hz frequency.

We further explored the mechanism underpinning the injectability of C8-ILD-NH_2_(**2**) hydrogel by studying its thixotropic properties (Figure 5f). The shear strain imposed on the gel started at 0.5 min and 0.1%, which the gel could tolerate, but was changed at 2 min to 200%, which broke the gel. The shear strain was reduced to 0% at 6.3 min and then returned to 0.1% at 7.5 min. The G′ and G″ curves switched in approximately 10 s from G′ > G″ to G″ < G′ and the values of both G′ and G″ dropped from approximately 3000–12,000 Pa to approximately 5–50 Pa upon changing the strain from 0.1% to 200%. These changes revealed the rapid shear-thinning property of the C8-ILD-NH_2_(**2**) hydrogel, as the gel was liquefied by the increased shear strain. Moreover, the G′ and G″ values completely switched back to the values observed at 0.1% strain at 0.5–2 min, when the shear strain was returned to 0.1% at 7.5 min, indicating the complete regeneration of the gel. The shear-thinning and regenerative properties of the C8-ILD-NH_2_(**2**) hydrogel provided mechanistic insights into its potential for injectability and were confirmed as completely repeatable by a thixotropic test repeated at 18 min (Figure 5f).

### 3.3. Fibrous Networks in Hydrogels Self-Assembled from a Selected Cn Fatty Acid–AA1–AA2–D Conjugate: The Study Using FESEM

**Figure 6:**
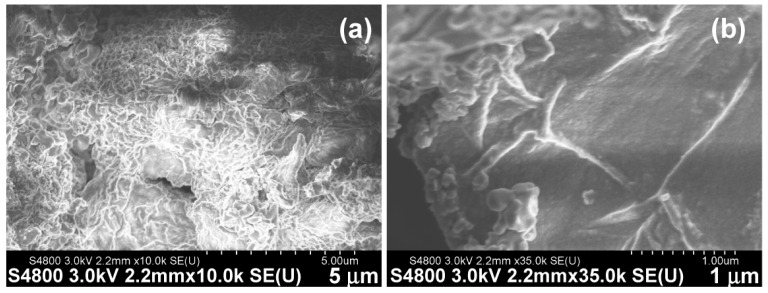
Surface structures of the fibrous networks from freeze-dried C8-ILD-NH_2_(**2**) hydrogel were observed using field emission scanning electron microscopy at different magnifications. Secondary electrons (SEs) were detected using an upper detector (U), with a working distance of 2.2 mm and acceleration voltage of −3.0 kV. (**a**) Image at ×10,000 (**b**) Image at ×35,000.

To understand the hydrogel network structures formed from Cn fatty acid–AA1–AA2–D conjugates, we performed an FESEM study of the representative one, namely the C8-ILD-NH_2_(**2**) hydrogel. FESEM showed the upper layer of fibrous networks under ×10,000 magnification (Figure 6a). These networks were polygonal. The SEM image of the hydrogel under ×35,000 magnification revealed the networks comprising fibrils with lengths > 1 µm comparable to those reported by other researchers (Figure 6b) [42,43]. There is a high propensity for C8-ILD-NH_2_(**2**) molecules to form fibrils through intermolecular hydrogen bonds between N-H (hydrogen-bonding donor) and C=O (hydrogen-bonding acceptor) groups, as it contains four N-H groups and five C=O groups to form hydrogen bonds along its backbone (Figure 2a). Of note, the nonpolar hydrocarbon side chains of I (isoleucine) and L (leucine) as well as the hydrocarbon tail of C8 acid in (**2**) can interact with the counterpart groups of another (**2**) through hydrophobic force, which also contributes to the formation of a hydrogel network [66].

### 3.4. The Hydrogel Formed from a Selected Cn Fatty Acid–AA1–AA2–D Conjugate Accelerated the Healing of Third-Degree Burn Wounds

**Figure 7:**
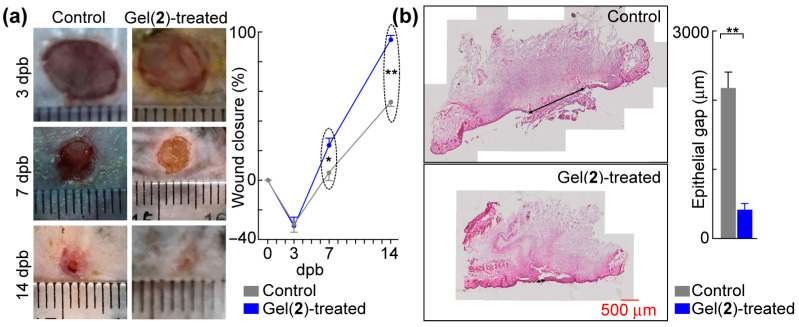
C8-ILD-NH_2_(**2**) hydrogel accelerated the healing of third-degree burn wounds in mice. (**a**) Left, typical photographs of wounds. The thin transparent ruler in the focal plate was used as the scale bar. Right, hydrogel formed by (**2**) promoted wound closure. (**b**) Hydrogel formed by (**2**) accelerated epithelialization. Left, representative micro-images of hematoxylin/eosin-stained wound sections (top: control; bottom: Gel(2)-treated); right, epithelial gaps. Results are presented as mean ± SEM with * *p* < 0.05, ** *p* < 0.01 (*t*-test).

C8-ILD-NH_2_(**2**) was selected as the initial candidate for burn-wound treatment from the 13 novel Cn fatty acid–AA1–AA2–D gelable conjugates synthesized in this study (Table 1) because it formed a hydrogel faster and with greater elasticity than the other 13 conjugates. This selection was further supported by our finding that the hydrogel formed from (**2**) had injectable, shear-thinning, and rapid re-gelling properties in saline at a broad pH range (pH 2–8) compatible with wound treatment. The C8-ILD-NH_2_(**2**) hydrogel (3% in PBS and 30 µL/wound) was injected to fill necrotic skin excised by third-degree burn wounds in 18-month-old *C57BL/6J* mice at day 2 dpb. The wound sizes were measured during the course of healing. The wound closure at 7 dpb, at 23.4% in the C8-ILD-NH_2_(**2**) gel-treated group, was significantly higher than the 5.2% closure for the control group (*p* < 0.05) (Figure 7a,b). At 14 dpb, the difference in wound closure was more significant (*p* < 0.01), as the wounds were 94.7% closed in the hydrogel-treated group compared to 52.9% in the control. Wound closure is a crucial step in the early healing phase, as it forms a barrier against infection and water loss while participating in and supporting other healing processes. The Tegaderm film dressing can markedly reduce the skin contraction as demonstrated by prior reports [50,51,52]. This forces the wound closure more by re-epitheliazation. Our method of film application is likely to render the contraction control more effective and durable than those in references [50,51,52]. The promotion of wound closure by the C8-ILD-NH_2_(**2**) gel demonstrated its prohealing properties and should act on wound re-epithelialization as well as contraction [67].

The acceleration of wound healing by the C8-ILD-NH_2_(**2**) hydrogel was confirmed with a histological analysis of the wound re-epithelialization (Figure 7b). Effective wound healing is characterized by the prompt regrowth of neoepithelium and regeneration of connective tissue by fibroblasts in the wound region [68]. Figure 7b shows the regeneration of epithelia from the wound edges, which resulted in a wound area with a higher degree of re-epithelialization in the C8-ILD-NH_2_(**2**) gel-treated wounds than in the nongel control wounds (411.4 vs. 2175.9 µm, *p* < 0.01). The control wounds also formed a scab on the wound surface (Figure 7b, left). Thus, the C8-ILD-NH_2_(**2**) hydrogel significantly promoted the re-epithelialization of third-degree burn wounds in *C57/BL6j* mice.

In summary, the C8-ILD-NH_2_(**2**) hydrogel can accelerate wound healing. No differences were found between the hydrogel-treated group and the PBS-treated control group in terms of mouse behavior, including drinking, foraging, grooming, and eating, or in terms of bodyweight or the size and weight of the liver, kidneys, and spleen. This hydrogel did not show systematic or local toxicity during the wound healing test. Our in vivo observations suggest that the application of hydrogel to wounds did not elicit any toxic or adverse effects on mouse health. Our pilot study of the C8-ILD-NH_2_(**2**) hydrogel for wound treatment has provided preclinical data for the further development of Cn fatty acid–AA1–AA2–D conjugate-based biomaterials for treating burn wounds in humans.

Notably, at 3 dpb, the wound sizes in both groups were actually larger than the initial size (6 mm in diameter) of the burn wounds created at 0 dpb, whereas the closure (%) at 3 dpb was negative (Figure 7a). The 6 mm heated rod used for burning cauterized/destroyed the 6-mm-diameter full-thickness skin underneath it; however, it also injured the tissue around its perimeter, causing the burn-wound margin to expand at 3 dpb, even though the necrotic tissue created by burning had already been debrided by conducting the 6 mm diameter full-thickness excision at 2 dpb. No differences were noted in wound size or closure at 3 dpb between the hydrogel-treated and control groups, which suggests that the hydrogel might be unable to rescue all damaged tissue or cells that were already destined to die. However, the promotion of wound closure and re-epithelialization by the hydrogel (Figure 7) may reflect its action in promoting growth from surrounding non-injured tissues and cells and/or its ability to partially rescue heat-injured tissue/cells [30,31]. The hydrogel may also affect wound vascularization, inflammation, and late-phase remodeling [30,31]. The hydrogels reported here may also have effects on bacteria and other microbes [4]. These possibilities are beyond the scope of this paper and will be examined in our future studies.

### 3.5. Ongoing and Future Directions

This report demonstrated the promising potential of novel gelable analogs formed by conjugating fatty acids and AA1–AA2–D tripeptides for wound treatment. We are currently screening other novel analogs in which D is replaced by other amino acids, such as glutamic acid, lysine, arginine, or histidine, with electrically charged side chains, and in which the Cn fatty acid is replaced by other fatty acids and their derivatives to generate biomaterials that can be used for better therapeutics. The Cn fatty acid–AA1–AA2–D analogs likely self-assembled into fibrous structures that further interacted with each other to form the hydrogel network. This prediction will be further tested in transmission electron microscopy and circular dichroism studies in the future. We also need to conduct all these studies on the other novel hydrogels that we synthesized in this pilot study.

## 4. Conclusions

We conducted a rational design and organic synthesis of molecular structures capable of forming prohealing hydrogels using two Fmoc/tBu-based SPPS routes, and we identified 13 novel conjugates of saturated fatty acid–aminoacid1–amonoacid2–asparatic acid that can form hydrogels under wound-compatible conditions. The compounds were purified, and analyzed using LCMS and ^1^H and ^13^C NMR spectroscopic techniques. Among them, C8 acid-ILD-NH_2_(**2**) was the best in terms of hydrogelation. Our results further revealed that C8 acid-ILD-NH_2_(**2**), in media resembling the pathophysiological wound condition, has desirable injectability, shear-thinning, and re-gelation features, as well as wound-compatible mechanical properties, that make this compound valuable in wound treatment. The C8 acid-ILD-NH_2_(**2**) hydrogel can markedly accelerate the healing of third-degree burn wounds. These results demonstrated the potential of the Cn fatty acid–AA1–AA2–D molecular template to form hydrogels capable of effectively promoting wound healing.

## Figures and Tables

**Table 1:** Novel fatty acid–peptide covalently bonded conjugates developed in this report: a brief of their chemical structures, MS/MS ions, and hydrogelation features.

Compound #	Structure ^(a)^	MS/MS Ions, *m*/*z* ^(b)^	Gelation Feature ^(c)^	Gelation Time (min)
1	H-ILD-NH_2_	357.2 (M − H), 295.3, 268.3, 242.3, 225.2	Not gelate	NA
2	C8-ILD-NH_2_	See Section 3.1.1	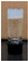	2
3	C12-ILD-NH_2_	539.5 (M − H), 522.1, 496.3, 424.2, 406.8, 296.2	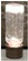	6
4	C16-ILD-NH_2_	595.5 (M − H), 578.5, 552.5, 481.4, 463.4, 351.5	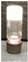	10
5	C8-ILD-OH	484.5 (M − H), 440.4, 394.4, 369.5, 350.5, 325.6, 130.2	Not gelate	NA
6	C12-ILD-OH	540.4 (M − H), 496.4, 425.2, 406.4, 294.6, 198.4	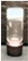	7
7	C14-ILD-OH	568.5 (M − H), 524.4, 453.6, 434.5, 409.6, 339.7, 322.7	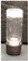	10
8	C16-ILD-OH	596.5 (M − H), 578.4, 481.4, 462.4, 437.3, 350.5	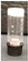	15
9	C8-IAD-NH_2_	441.4 (M − H), 352.2, 326.5, 308.2, 200.3, 182.3	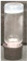	2
10	C8-IVD-NH_2_	469.5 (M − H), 425.5, 380.5, 354.4, 336.1, 281.6	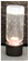	3
11	C8-ALD-NH_2_	441.4 (M − H), 326.4, 308.4, 200.0, 182.3, 165.2	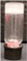	3
12	C8-LLD-NH_2_	483.4 (M − H), 421.6, 368.5, 350.5, 295.7, 197.4, 165.2	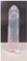	5
13	C8-VLD-NH_2_	469.4 (M − H), 451.4, 425.5, 380.4, 354.5, 336.4, 281.5, 193.3	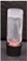	6
14	C8-IID-NH_2_	483.5 (M − H), 421.5, 368.4, 350.4, 295.5, 197.2, 165.3	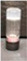	5
15	C8-LID-NH_2_	483.5 (M − H), 368.4, 350.4, 295.4, 181.7, 165.1	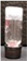	5

Notes: ^(a)^ In the structure column, C8, C12, C14, and C16 denote the corresponding carbon chain length; letters I, L, D, A, and V denote amino acids; I—isoleucine, L—leucine, D—aspartic acid, A—alanine, V—valine; -NH_2_: C-terminus amide group; and -OH: C-terminus carboxylic acid group; ^(b)^ MS/MS ion acquired by LC-MS/MS, single charged (z = 1, *m*/*z* = Daltons); ^(c)^ conjugate: 1.5% *w*/*v* in PBS saline, pH 6.5–7.8. NA: not applicable.

## Data Availability

Data is contained within the article.

## Supplementary Materials

The following supplementary information can be downloaded at: https://www.mdpi.com/article/10.3390/biom14010094/s1, Figures S1–S27. ^1^H, ^13^C, and ^13^C DEPT-135 NMR spectra of compounds **6**–**14**. Video S1: Quick hydrogelation of compound C8 acid-ILD–NH_2_(**2**).

## References

[1] Zhang X., Wei X., Liu L., Marti G.P., Ghanamah M.S., Arshad M.J., Strom L., Spence R., Jeng J., Milner S. (2010). Association of increasing burn severity in mice with delayed mobilization of circulating angiogenic cells. Arch. Surg..

[2] Lewis C.J. (2013). Stem cell application in acute burn care and reconstruction. J. Wound Care.

[3] Rogers A.D., Jeschke M.G. (2016). Managing severe burn injuries: Challenges and solutions in complex and chronic wound care. Chrnonic Wound Care Manag. Res..

[4] Murphy P.S., Evans G.R. (2012). Advances in wound healing: A review of current wound healing products. Plast. Surg. Int..

[5] Huang C., Yuan W., Chen J., Wu L.P., You T. (2023). Construction of Smart Biomaterials for Promoting Diabetic Wound Healing. Molecules.

[6] Shi Q., Qian Z., Liu D., Sun J., Wang X., Liu H., Xu J., Guo X. (2017). GMSC-Derived Exosomes Combined with a Chitosan/Silk Hydrogel Sponge Accelerates Wound Healing in a Diabetic Rat Skin Defect Model. Front. Physiol..

[7] Park J.S., An S.J., Jeong S.I., Gwon H.J., Lim Y.M., Nho Y.C. (2017). Chestnut Honey Impregnated Carboxymethyl Cellulose Hydrogel for Diabetic Ulcer Healing. Polymers.

[8] Zhang Y., Zheng Y., Shu F., Zhou R., Bao B., Xiao S., Li K., Lin Q., Zhu L., Xia Z. (2022). In situ-formed adhesive hyaluronic acid hydrogel with prolonged amnion-derived conditioned medium release for diabetic wound repair. Carbohydr. Polym..

[9] Shah S.A., Sohail M., Khan S.A., Kousar M. (2021). Improved drug delivery and accelerated diabetic wound healing by chondroitin sulfate grafted alginate-based thermoreversible hydrogels. Mater. Sci. Eng. C Mater. Biol. Appl..

[10] Kawabata S., Kanda N., Hirasawa Y., Noda K., Matsuura Y., Suzuki S., Kawai K. (2018). The Utility of Silk-elastin Hydrogel as a New Material for Wound Healing. Plast. Reconstr. Surg. Glob. Open.

[11] Wu S., Yang Y., Wang S., Dong C., Zhang X., Zhang R., Yang L. (2022). Dextran and peptide-based pH-sensitive hydrogel boosts healing process in multidrug-resistant bacteria-infected wounds. Carbohydr. Polym..

[12] Certelli A., Valente P., Uccelli A., Grosso A., Di Maggio N., D’Amico R., Briquez P.S., Hubbell J.A., Wolff T., Gurke L. (2021). Robust Angiogenesis and Arteriogenesis in the Skin of Diabetic Mice by Transient Delivery of Engineered VEGF and PDGF-BB Proteins in Fibrin Hydrogels. Front. Bioeng. Biotechnol..

[13] Rezvanian M., Amin M., Ng S.F. (2016). Development and physicochemical characterization of alginate composite film loaded with simvastatin as a potential wound dressing. Carbohydr. Polym..

[14] Qian B., Li J., Guo K., Guo N., Zhong A., Yang J., Wang J., Xiao P., Sun J., Xiong L. (2021). Antioxidant biocompatible composite collagen dressing for diabetic wound healing in rat model. Regen. Biomater..

[15] Ghobril C., Grinstaff M.W. (2015). The chemistry and engineering of polymeric hydrogel adhesives for wound closure: A tutorial. Chem. Soc. Rev..

[16] Pan Z., Ye H., Wu D. (2021). Recent advances on polymeric hydrogels as wound dressings. APL Bioeng..

[17] Kirker K.R., Luo Y., Nielson J.H., Shelby J., Prestwich G.D. (2002). Glycosaminoglycan hydrogel films as bio-interactive dressings for wound healing. Biomaterials.

[18] Boucard N., Viton C., Agay D., Mari E., Roger T., Chancerelle Y., Domard A. (2007). The use of physical hydrogels of chitosan for skin regeneration following third-degree burns. Biomaterials.

[19] Kiyozumi T., Kanatani Y., Ishihara M., Saitoh D., Shimizu J., Yura H., Suzuki S., Okada Y., Kikuchi M. (2007). The effect of chitosan hydrogel containing DMEM/F12 medium on full-thickness skin defects after deep dermal burn. Burns.

[20] Kim K.L., Han D.K., Park K., Song S.H., Kim J.Y., Kim J.M., Ki H.Y., Yie S.W., Roh C.R., Jeon E.S. (2009). Enhanced dermal wound neovascularization by targeted delivery of endothelial progenitor cells using an RGD-g-PLLA scaffold. Biomaterials.

[21] Madsen J., Armes S.P., Bertal K., Lomas H., Macneil S., Lewis A.L. (2008). Biocompatible wound dressings based on chemically degradable triblock copolymer hydrogels. Biomacromolecules.

[22] Shepherd J., Sarker P., Rimmer S., Swanson L., MacNeil S., Douglas I. (2011). Hyperbranched poly(NIPAM) polymers modified with antibiotics for the reduction of bacterial burden in infected human tissue engineered skin. Biomaterials.

[23] Balakrishnan B., Mohanty M., Umashankar P.R., Jayakrishnan A. (2005). Evaluation of an in situ forming hydrogel wound dressing based on oxidized alginate and gelatin. Biomaterials.

[24] Altman A.M., Matthias N., Yan Y., Song Y.H., Bai X., Chiu E.S., Slakey D.P., Alt E.U. (2008). Dermal matrix as a carrier for in vivo delivery of human adipose-derived stem cells. Biomaterials.

[25] Simpson D., Liu H., Fan T.H., Nerem R., Dudley S.C. (2007). A tissue engineering approach to progenitor cell delivery results in significant cell engraftment and improved myocardial remodeling. Stem Cells.

[26] Kutschka I., Chen I.Y., Kofidis T., Arai T., von Degenfeld G., Sheikh A.Y., Hendry S.L., Pearl J., Hoyt G., Sista R. (2006). Collagen matrices enhance survival of transplanted cardiomyoblasts and contribute to functional improvement of ischemic rat hearts. Circulation.

[27] Jonker A.M., Löwik D.W.P.M., van Hest J.C.M. (2012). Peptide- and Protein-Based Hydrogels. Chem. Mater..

[28] Branco M.C., Schneider J.P. (2009). Self-assembling materials for therapeutic delivery. Acta Biomater..

[29] Collier J.H., Rudra J.S., Gasiorowski J.Z., Jung J.P. (2010). Multi-component extracellular matrices based on peptide self-assembly. Chem. Soc. Rev..

[30] Yang Z., Xu K., Wang L., Gu H., Wei H., Zhang M., Xu B. (2005). Self-assembly of small molecules affords multifunctional supramolecular hydrogels for topically treating simulated uranium wounds. Chem. Commun..

[31] Yang Z., Liang G., Ma M., Abbah A.S., Lu W.W., Xu B. (2007). D-glucosamine-based supramolecular hydrogels to improve wound healing. Chem. Commun..

[32] Tenidis K., Waldner M., Bernhagen J., Fischle W., Bergmann M., Weber M., Merkle M.L., Voelter W., Brunner H., Kapurniotu A. (2000). Identification of a penta- and hexapeptide of islet amyloid polypeptide (IAPP) with amyloidogenic and cytotoxic properties. J. Mol. Biol..

[33] Reches M., Porat Y., Gazit E. (2002). Amyloid fibril formation by pentapeptide and tetrapeptide fragments of human calcitonin. J. Biol. Chem..

[34] Hsieh M.C., Liang C., Mehta A.K., Lynn D.G., Grover M.A. (2017). Multistep Conformation Selection in Amyloid Assembly. J. Am. Chem. Soc..

[35] Pappas C.G., Shafi R., Sasselli I.R., Siccardi H., Wang T., Narang V., Abzalimov R., Wijerathne N., Ulijn R.V. (2016). Dynamic peptide libraries for the discovery of supramolecular nanomaterials. Nat. Nanotechnol..

[36] Tang J.D., Mura C., Lampe K.J. (2019). Stimuli-Responsive, Pentapeptide, Nanofiber Hydrogel for Tissue Engineering. J. Am. Chem. Soc..

[37] Yadav N., Kumar U., Roopmani P., Krishnan U.M., Sethuraman S., Chauhan M.K., Chauhan V.S. (2022). Ultrashort Peptide-Based Hydrogel for the Healing of Critical Bone Defects in Rabbits. ACS Appl. Mater. Interfaces.

[38] Hauser C.A., Deng R., Mishra A., Loo Y., Khoe U., Zhuang F., Cheong D.W., Accardo A., Sullivan M.B., Riekel C. (2011). Natural tri- to hexapeptides self-assemble in water to amyloid β-type fiber aggregates by unexpected α-helical intermediate structures. Proc. Natl. Acad. Sci. USA.

[39] Owen S.C., Shoichet M.S. (2010). Design of three-dimensional biomimetic scaffolds. J. Biomed. Mater. Res. A.

[40] Wang Y., Mithieux S.M., Kong Y., Wang X.Q., Chong C., Fathi A., Dehghani F., Panas E., Kemnitzer J., Daniels R. (2015). Tropoelastin incorporation into a dermal regeneration template promotes wound angiogenesis. Adv. Healthc. Mater..

[41] Panahi Y., Gharekhani A., Hamishehkar H., Zakeri-Milani P., Gharekhani H. (2019). Stomach-Specific Drug Delivery of Clarithromycin Using a Semi Interpenetrating Polymeric Network Hydrogel Made of Montmorillonite and Chitosan: Synthesis, Characterization and In Vitro Drug Release Study. Adv. Pharm. Bull..

[42] Reithofer M.R., Chan K., Lakshmanan A., Lam D.H., Mishra A., Gopalan B., Joshi M., Wanga S., Hauser C.A.E. (2014). Ligation of anti-cancer drugs to self-assembling ultrashort peptides by click chemistry for localized therapy. Chem. Sci..

[43] Zhu J., Han H., Ye T.T., Li F.X., Wang X.L., Yu J.Y., Wu D.Q. (2018). Biodegradable and pH Sensitive Peptide Based Hydrogel as Controlled Release System for Antibacterial Wound Dressing Application. Molecules.

[44] Percie du Sert N., Hurst V., Ahluwalia A., Alam S., Avey M.T., Baker M., Browne W.J., Clark A., Cuthill I.C., Dirnagl U. (2020). The ARRIVE guidelines 2.0: Updated guidelines for reporting animal research. J. Cereb. Blood Flow Metab..

[45] Sun G., Zhang X., Shen Y.I., Sebastian R., Dickinson L.E., Fox-Talbot K., Reinblatt M., Steenbergen C., Harmon J.W., Gerecht S. (2011). Dextran hydrogel scaffolds enhance angiogenic responses and promote complete skin regeneration during burn wound healing. Proc. Natl. Acad. Sci. USA.

[46] Hanjaya-Putra D., Shen Y.I., Wilson A., Fox-Talbot K., Khetan S., Burdick J.A., Steenbergen C., Gerecht S. (2013). Integration and regression of implanted engineered human vascular networks during deep wound healing. Stem Cells Transl. Med..

[47] Cash J.L., Bass M.D., Campbell J., Barnes M., Kubes P., Martin P. (2014). Resolution mediator chemerin15 reprograms the wound microenvironment to promote repair and reduce scarring. Curr. Biol..

[48] Zhang X., Liu L., Wei X., Tan Y.S., Tong L., Chang R., Ghanamah M.S., Reinblatt M., Marti G.P., Harmon J.W. (2010). Impaired angiogenesis and mobilization of circulating angiogenic cells in HIF-1α heterozygous-null mice after burn wounding. Wound Repair Regen..

[49] Zhang X., Sarkar K., Rey S., Sebastian R., Andrikopoulou E., Marti G.P., Fox-Talbot K., Semenza G.L., Harmon J.W. (2011). Aging impairs the mobilization and homing of bone marrow-derived angiogenic cells to burn wounds. J. Mol. Med..

[50] Chung T.Y., Peplow P.V., Baxter G.D. (2010). Laser photobiomodulation of wound healing in diabetic and non-diabetic mice: Effects in splinted and unsplinted wounds. Photomed. Laser Surg..

[51] Bae S.H., Bae Y.C., Nam S.B., Choi S.J. (2012). A skin fixation method for decreasing the influence of wound contraction on wound healing in a rat model. Arch. Plast. Surg..

[52] Yao Z., Huang Y., Luo G., Wu J., He W. (2014). A biological membrane-based novel excisional wound-splinting model in mice (with video). Burn. Trauma.

[53] Tian H., Lu Y., Shah S.P., Hong S. (2011). 14S,21R-Dihydroxydocosahexaenoic Acid Remedies Impaired Healing and Mesenchymal Stem Cell Functions in Diabetic Wounds. J. Biol. Chem..

[54] Tian H., Lu Y., Shah S.P., Hong S. (2011). Autacoid 14S,21R-dihydroxy-docosahexaenoic acid counteracts diabetic impairment of macrophage prohealing functions. Am. J. Pathol..

[55] Hong S., Alapure B.V., Lu Y., Tian H., Wang Q. (2014). 12/15-Lipoxygenase deficiency reduces densities of mesenchymal stem cells in the dermis of wounded and unwounded skin. Br. J. Dermatol..

[56] Hong S., Alapure B.V., Lu Y., Tian H., Wang Q. (2014). Immunohistological localization of endogenous unlabeled stem cells in wounded skin. J. Histochem. Cytochem..

[57] Hong S., Lu Y., Tian H., Alapure B.V., Wang Q., Bunnell B.A., Laborde J.M. (2014). Maresin-like lipid mediators are produced by leukocytes and platelets and rescue reparative function of diabetes-impaired macrophages. Chem. Biol..

[58] Hong S., Tian H., Lu Y., Laborde J.M., Muhale F.A., Wang Q., Alapure B.V., Serhan C.N., Bazan N.G. (2014). Neuroprotectin/protectin D1: Endogenous biosynthesis and actions on diabetic macrophages in promoting wound healing and innervation impaired by diabetes. Am. J. Physiol. Cell Physiol..

[59] Lu Y., Tian H., Hong S. (2010). Novel 14,21-dihydroxy-docosahexaenoic acids: Structures, formation pathways, and enhancement of wound healing. J. Lipid Res..

[60] Tian H., Lu Y., Shah S.P., Hong S. (2010). Novel 14S,21-dihydroxy-docosahexaenoic acid rescues wound healing and associated angiogenesis impaired by acute ethanol intoxication/exposure. J. Cell Biochem..

[61] Alapure B.V., Lu Y., He M., Chu C.C., Peng H., Muhale F., Brewerton Y.L., Bunnell B., Hong S. (2018). Accelerate Healing of Severe Burn Wounds by Mouse Bone Marrow Mesenchymal Stem Cell-Seeded Biodegradable Hydrogel Scaffold Synthesized from Arginine-Based Poly(ester amide) and Chitosan. Stem Cells Dev..

[62] Nie G., Hong K., Zhang E., Liu N., Wang M., Wang L., Zang Y. (2020). Fabrication of a porous chitosan/poly-(gamma-glutamic acid) hydrogel with a high absorption capacity by electrostatic contacts. Int. J. Biol. Macromol..

[63] Ghobril C., Charoen K., Rodriguez E.K., Nazarian A., Grinstaff M.W. (2013). A dendritic thioester hydrogel based on thiol-thioester exchange as a dissolvable sealant system for wound closure. Angew. Chem. Int. Ed. Engl..

[64] Jones E.M., Cochrane C.A., Percival S.L. (2015). The Effect of pH on the Extracellular Matrix and Biofilms. Adv. Wound Care.

[65] Schneider L.A., Korber A., Grabbe S., Dissemond J. (2007). Influence of pH on wound-healing: A new perspective for wound-therapy?. Arch. Dermatol. Res..

[66] Sathaye S., Zhang H., Sonmez C., Schneider J.P., MacDermaid C.M., Von Bargen C.D., Saven J.G., Pochan D.J. (2014). Engineering complementary hydrophobic interactions to control β-hairpin peptide self-assembly, network branching, and hydrogel properties. Biomacromolecules.

[67] Chen L., Mirza R., Kwon Y., DiPietro L.A., Koh T.J. (2015). The murine excisional wound model: Contraction revisited. Wound Repair. Regen..

[68] Guo S., Dipietro L.A. (2010). Factors affecting wound healing. J. Dent. Res..

