# Supplementary material for: Development of a Novel Covalently Bonded Conjugate of Caprylic Acid Tripeptide (Isoleucine–Leucine–Aspartic Acid) for Wound-Compatible and Injectable Hydrogel to Accelerate Healing
Source: Biomolecules. 2024 Jan 11;14(1):94. doi: 10.3390/biom14010094 (PMC10813153; doi:10.3390/biom14010094)
Supplement: Supplementary file 1 [file biomolecules-14-00094-s001.zip › biomolecules-2803356-supplementary.pdf]

Supplemental Figures

Title: Development of a Novel Covalently Bonded Conjugate of Caprylic acid-Tripeptide (Isoleucine-Leucine-Aspartic Acid) for Wound-compatible and Injectable Hydrogel to Accelerate Healing

Sachin B. Baravkar[1,*], Yan Lu[1,*], Abdul-Razak Masoud[1], Qi Zhao[2], Jibao He[3], Song Hong[1,4,¥]

[1] Neuroscience Center of Excellence, School of Medicine, L.S.U. Health, New Orleans, LA 70112, U.S.A.; ylu@lsuhsc.edu (Y.L.); amasou@lsuhsc.edu (A.-R.M.)

[2] NMR Laboratory, Department of Chemistry, Tulane University, New Orleans, LA 70115, U.S.A.; qzhao@tulane.edu

[3] Microscopy Laboratory, Tulane University, New Orleans, LA 70115, U.S.A.

[4] Department of Ophthalmology, School of Medicine, L.S.U. Health, New Orleans, LA 70112, U.S.A.

*These authors contributed equally to this work and share first authorship

¥ Corresponding author: Song Hong, PhD, email: shong@lsuhsc.edu; Tel: 504 605 9013

Figures S1 to S27. ^{1}H, ^{13}C, and ^{13}C DEPT 135 NMR spectra of compounds 6-14.

Figure number	NMR spectra of compound #	Page number
Figures S1-S3	^{1}H, ^{13}C, and ^{13}C DEPT 135 NMR spectra for compound **6**	S2-S3
Figures S4-S6	^{1}H, ^{13}C, and ^{13}C DEPT 135 NMR spectra for compound **7**	S3-S4
Figures S7-S9	^{1}H, ^{13}C, and ^{13}C DEPT 135 NMR spectra for compound **8**	S5-S6
Figures S10-S12	^{1}H, ^{13}C, and ^{13}C DEPT 135 NMR spectra for compound **9**	S6-S7
Figures S13-S15	^{1}H, ^{13}C, and ^{13}C DEPT 135 NMR spectra for compound **10**	S8-S9
Figures S16-S18	^{1}H, ^{13}C, and ^{13}C DEPT 135 NMR spectra for compound **11**	S9-S10
Figures S19-S21	^{1}H, ^{13}C, and ^{13}C DEPT 135 NMR spectra for compound **12**	S11-S12
Figures S22-S24	^{1}H, ^{13}C, and ^{13}C DEPT 135 NMR spectra for compound **13**	S12-S13
Figures S25-S27	^{1}H, ^{13}C, and ^{13}C DEPT 135 NMR spectra for compound **14**	S14-S15

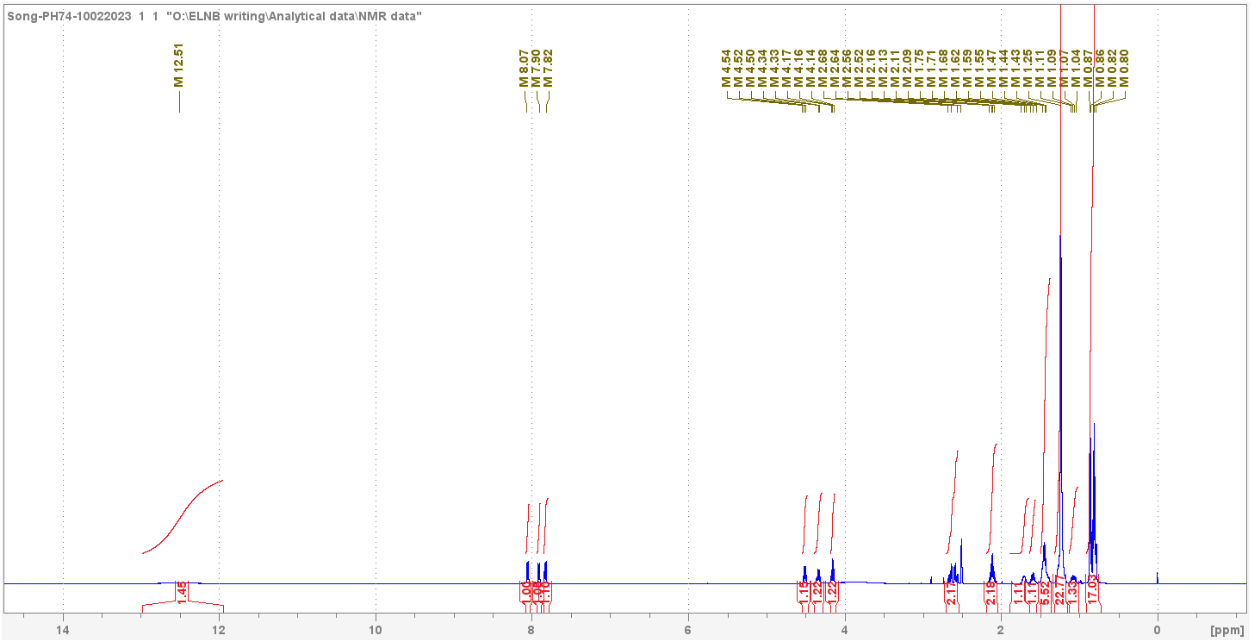

Figure S1: ^{1}H NMR of compound **6** in DMSO-d6.

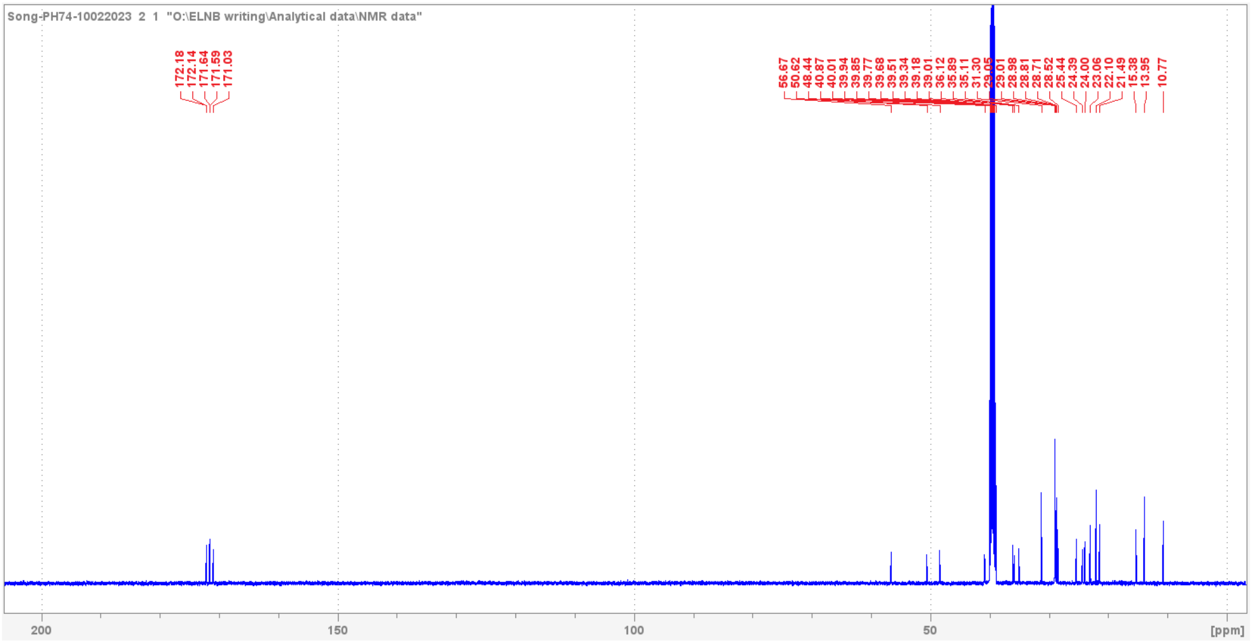

Figure S2: ^{13}C NMR of compound **6** in DMSO-d6.

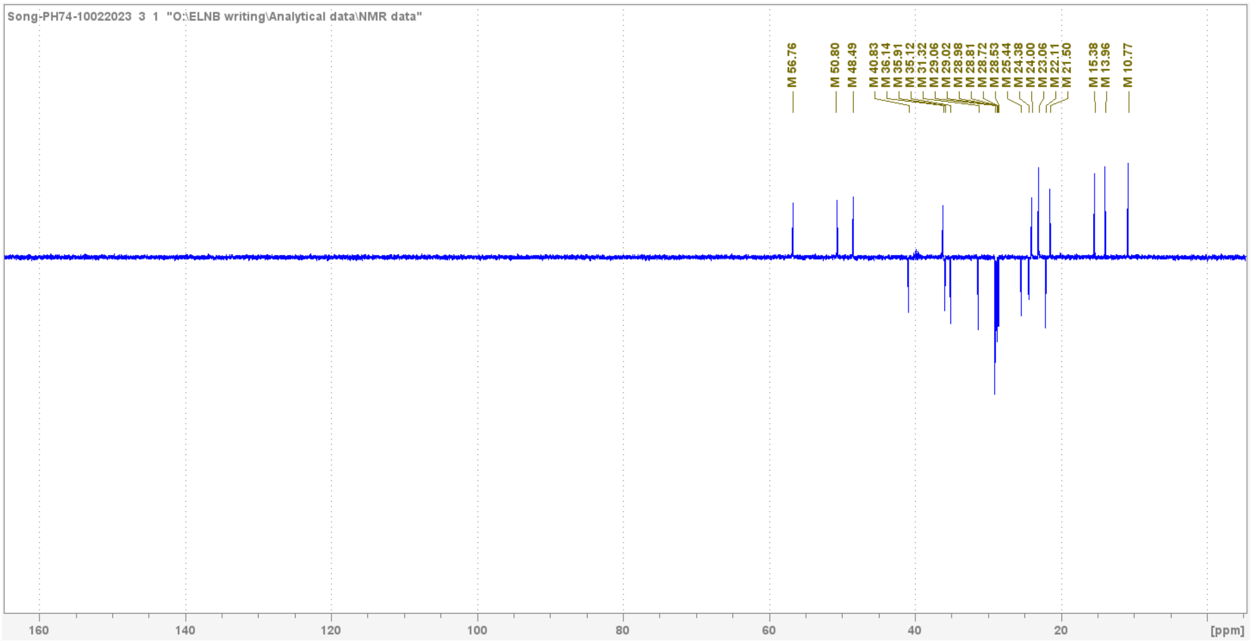

Figure S3: ^{13}C DEPT NMR of compound **6** in DMSO-d6.

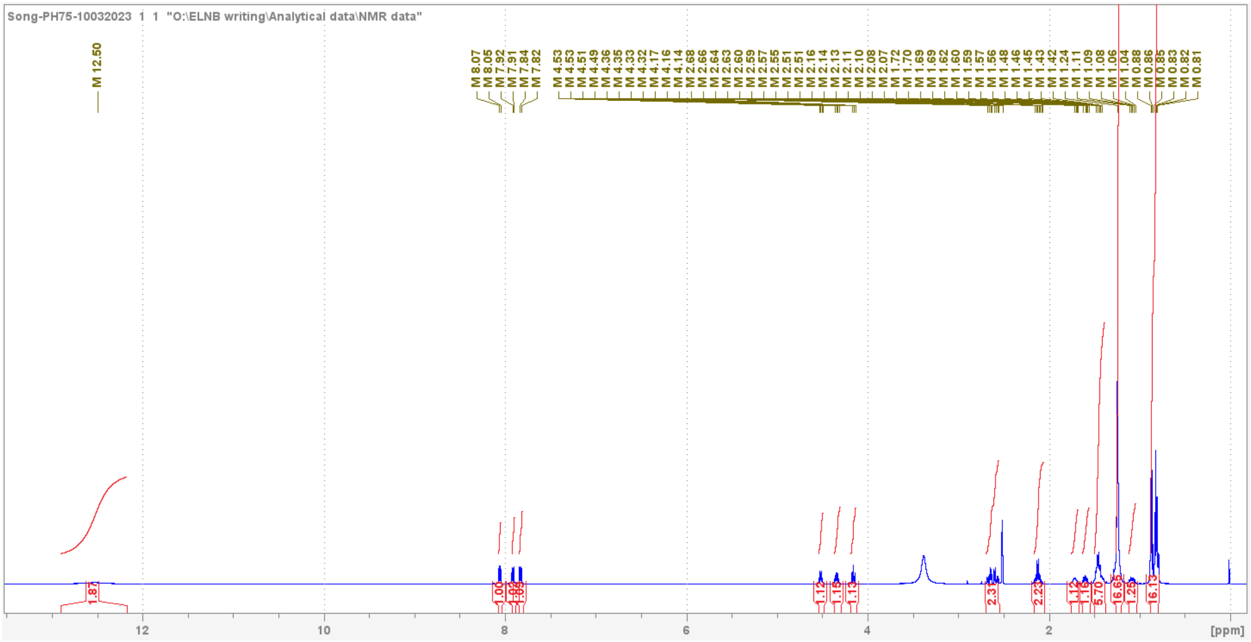

Figure S4: ^{1}H NMR of compound **7** in DMSO-d6.

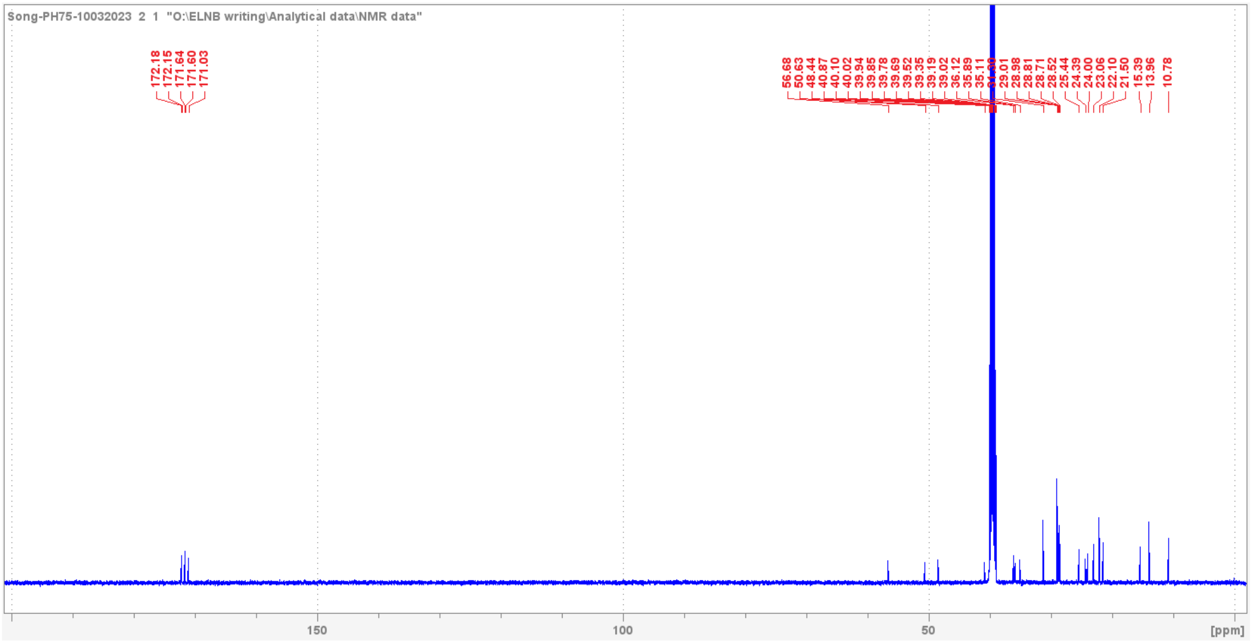

Figure S5: ^{13}C NMR of compound **7** in DMSO-d6.

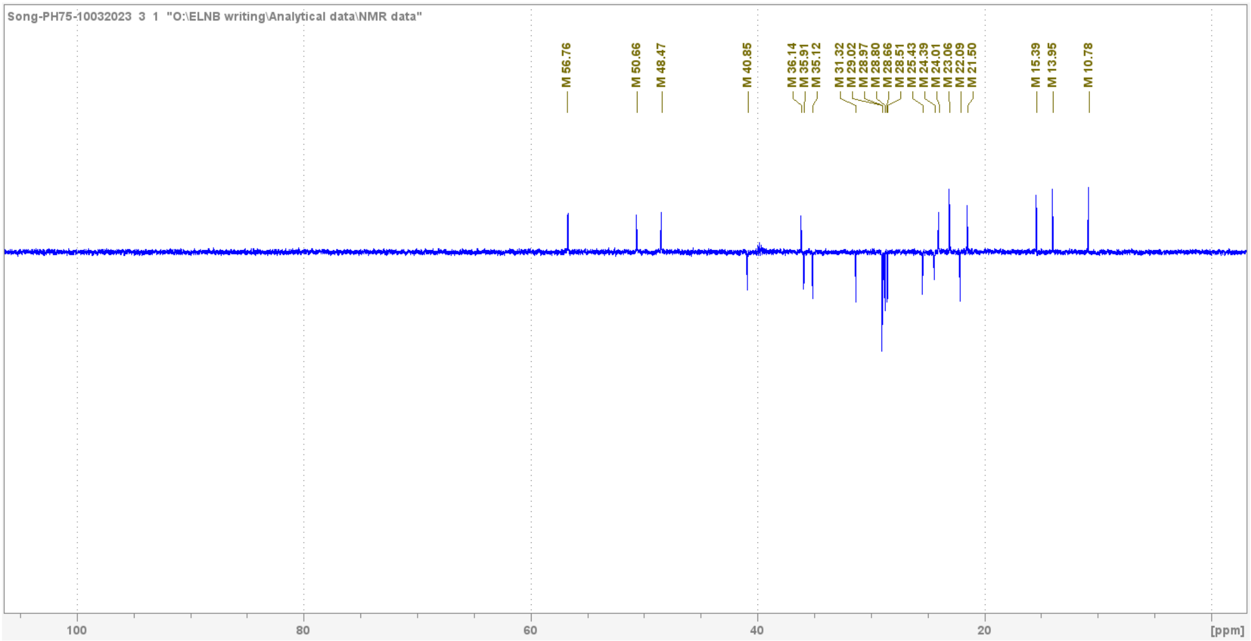

Figure S6: ^{13}C DEPT NMR of compound **7** in DMSO-d6.

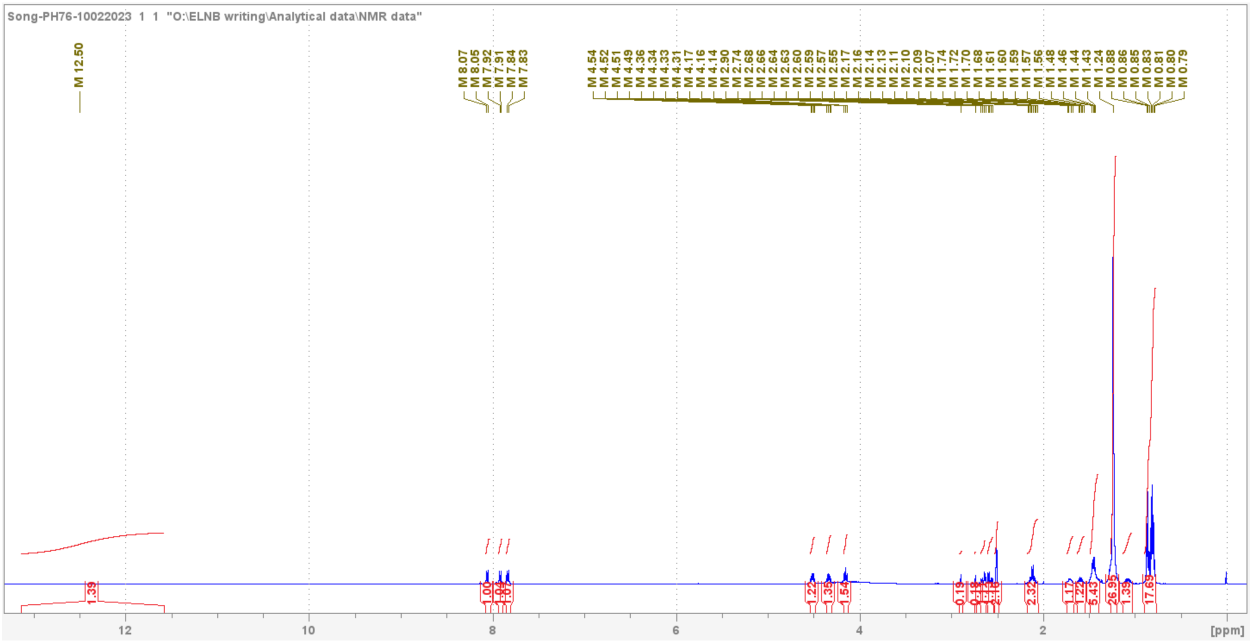

Figure S7: ^{1}H NMR of compound **8** in DMSO-d6.

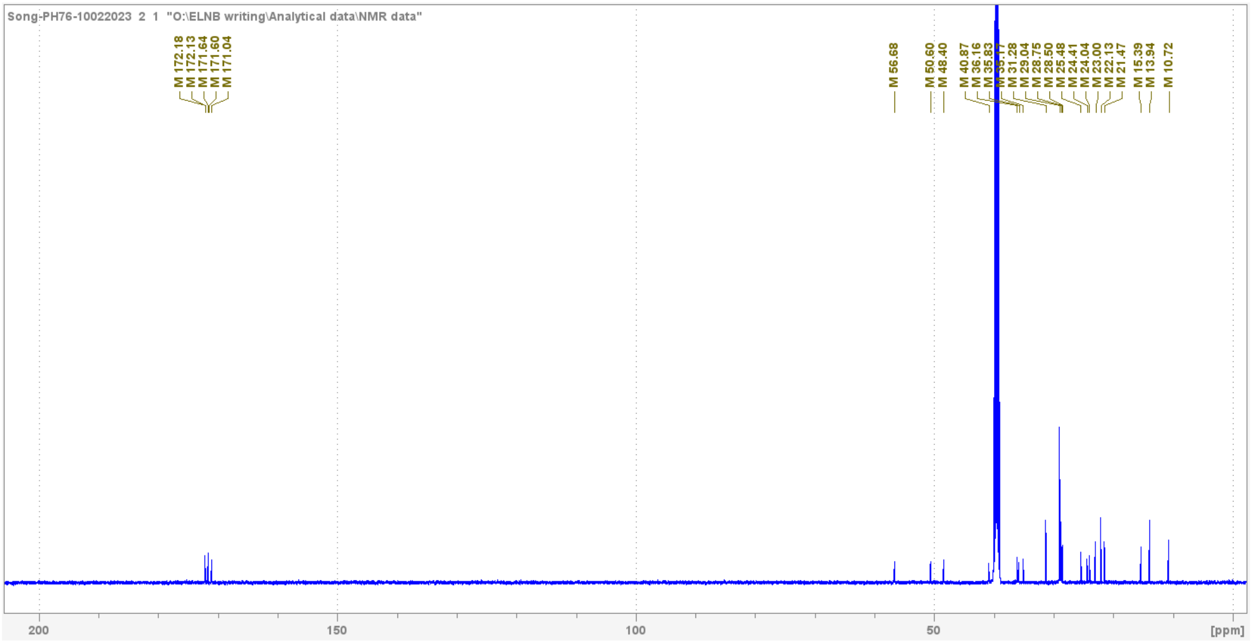

Figure S8: ^{13}C NMR of compound **8** in DMSO-d6.

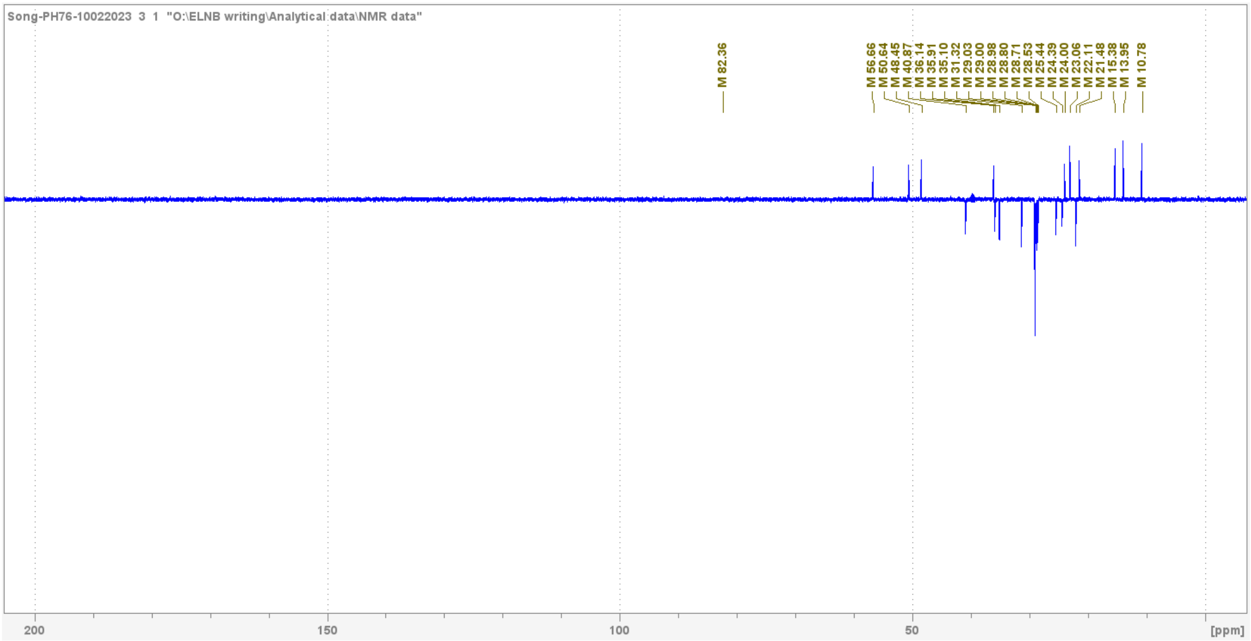

Figure S9: ^{13}C DEPT NMR of compound **8** in DMSO-d6.

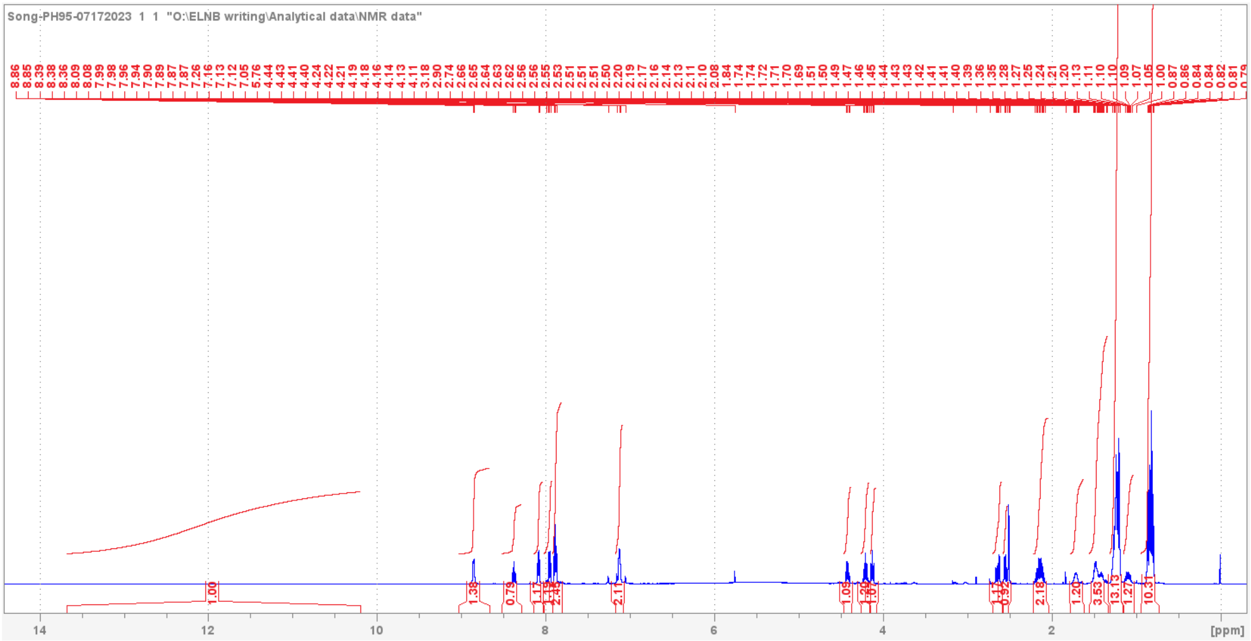

Figure S10: ^{1}H NMR of compound **9** in DMSO-d6.

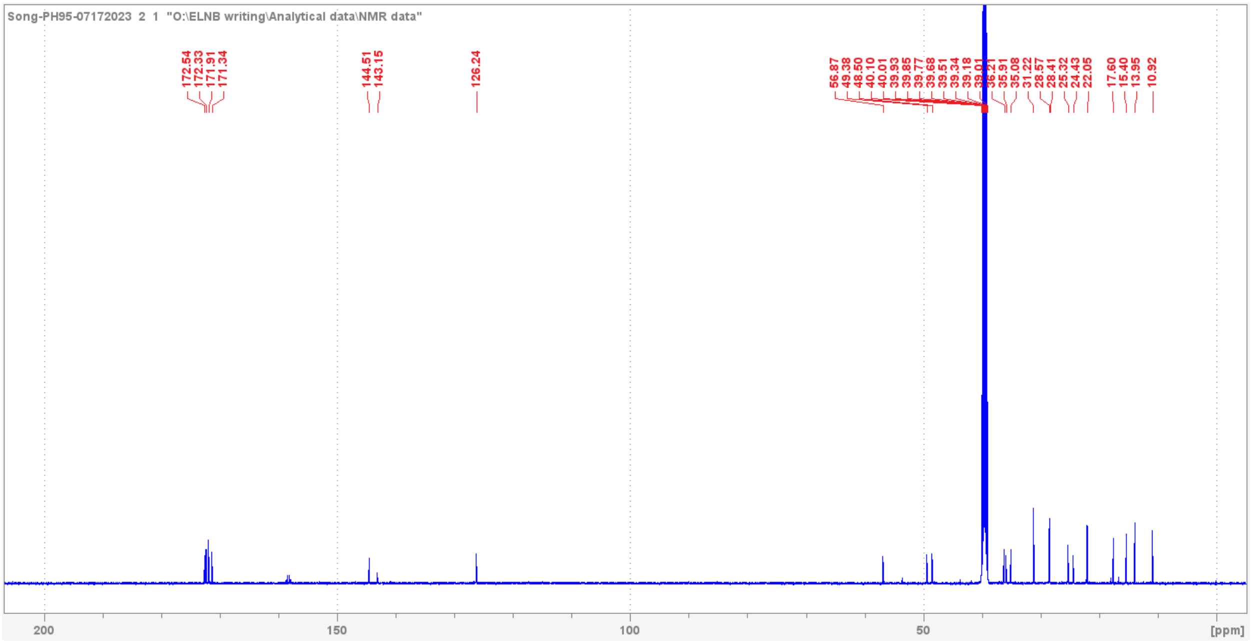

Figure S11: ^{13}C NMR of compound **9** in DMSO-d6.

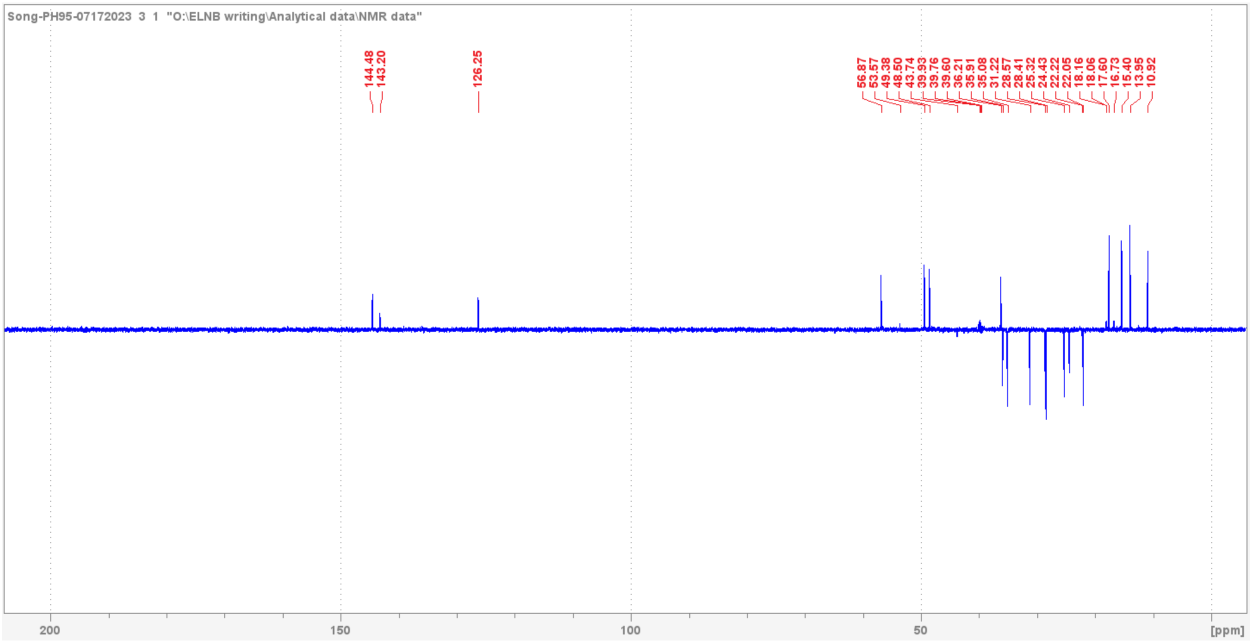

Figure S12: ^{13}C DEPT NMR of compound **9** in DMSO-d6.

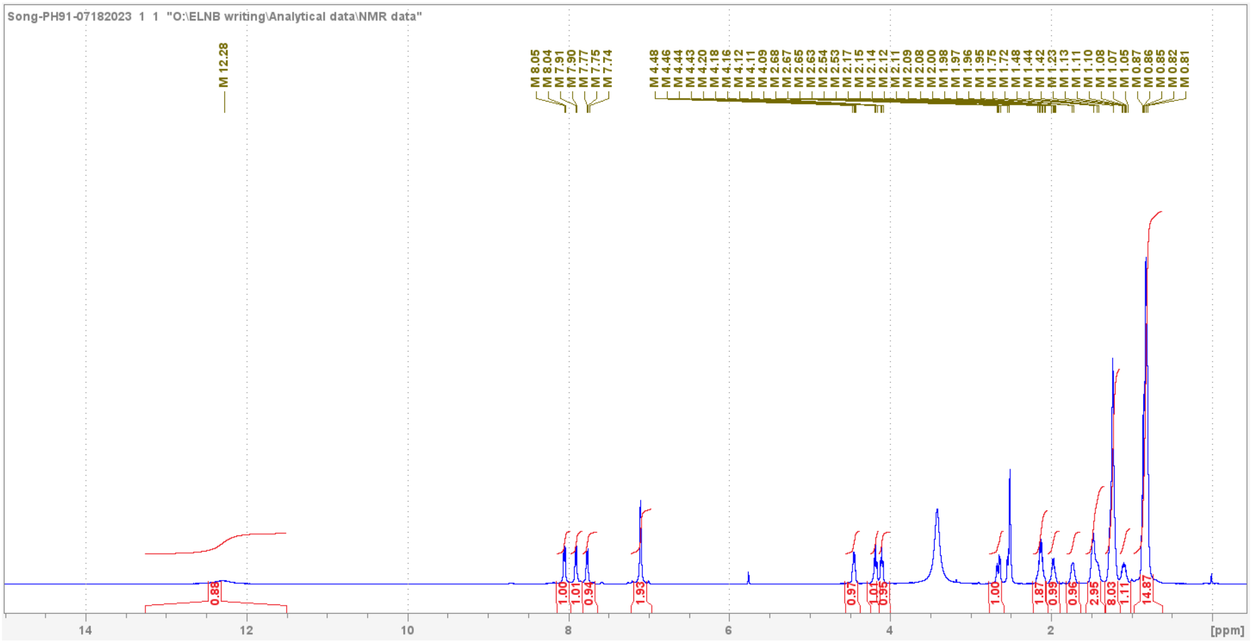

Figure S13: ^{1}H NMR of compound **10** in DMSO-d6.

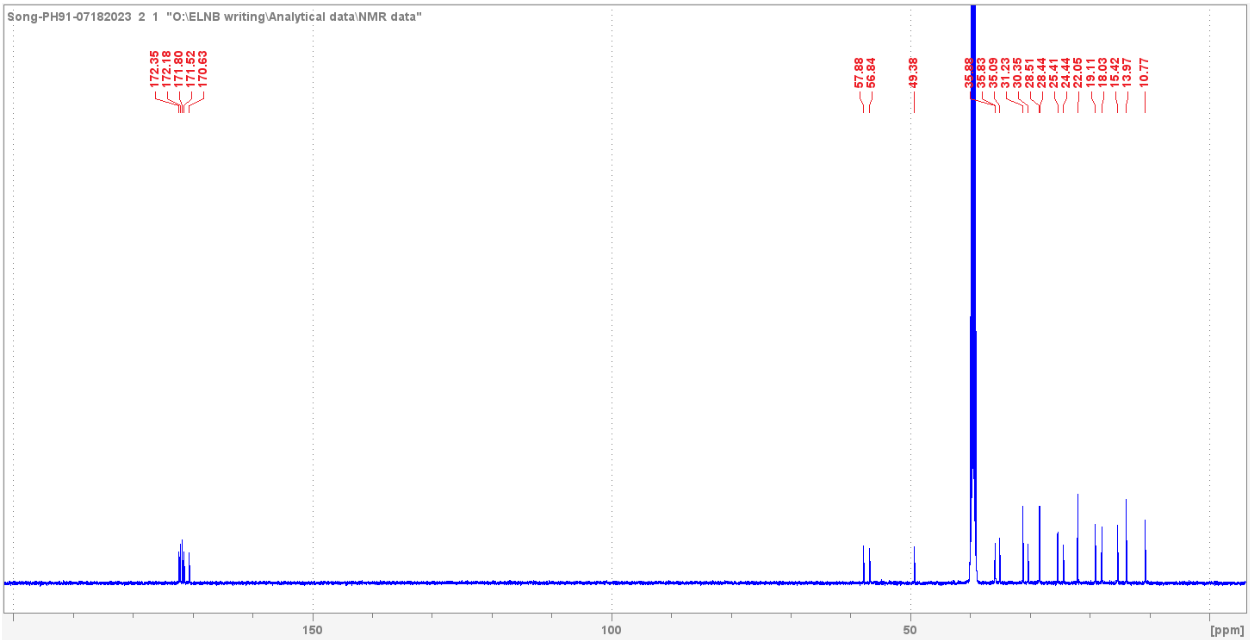

Figure S14: ^{13}C NMR of compound **10** in DMSO-d6.

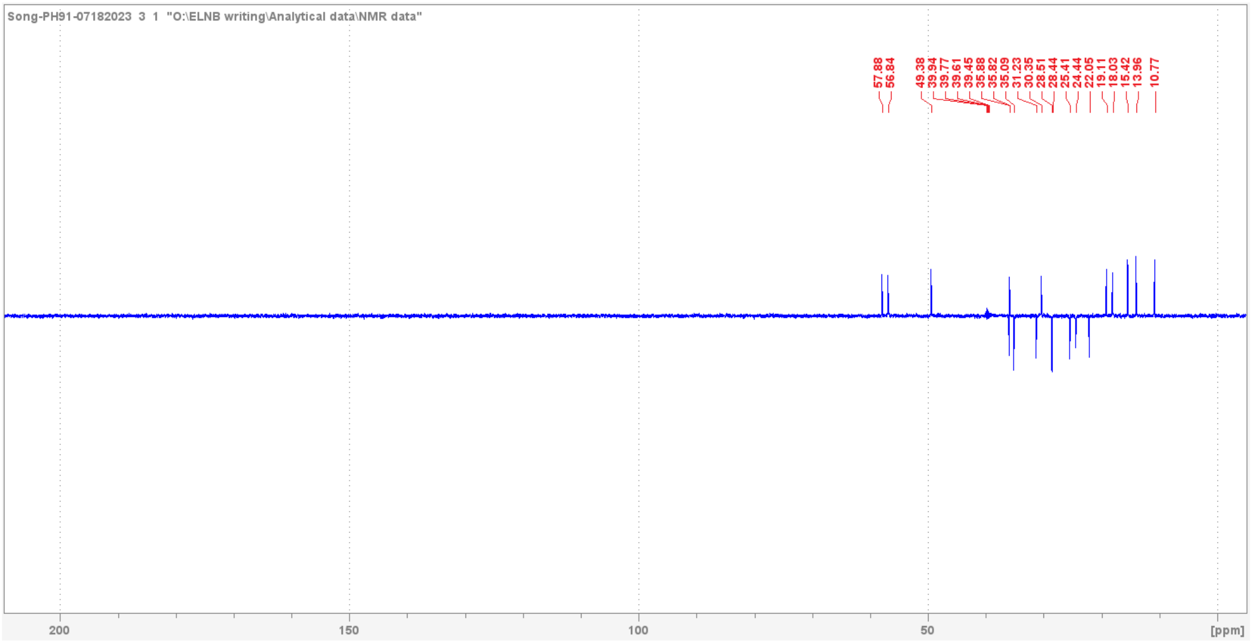

Figure S15: ^{13}C DEPT NMR of compound **10** in DMSO-d6.

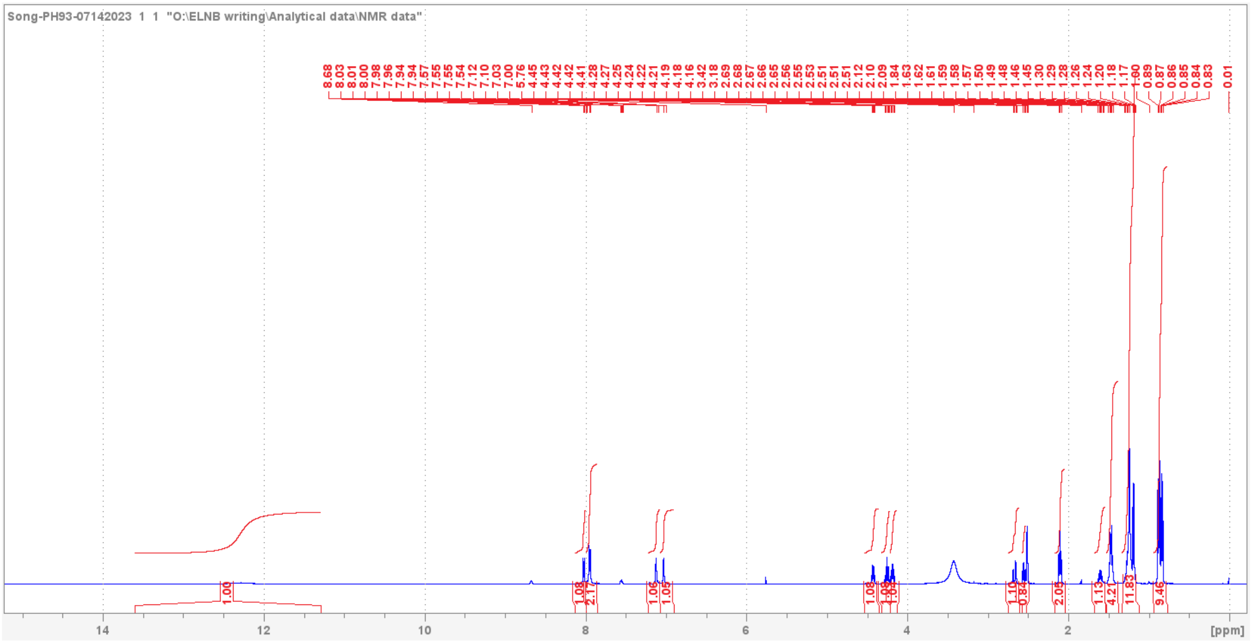

Figure S16: ^{1}H NMR of compound **11** in DMSO-d6.

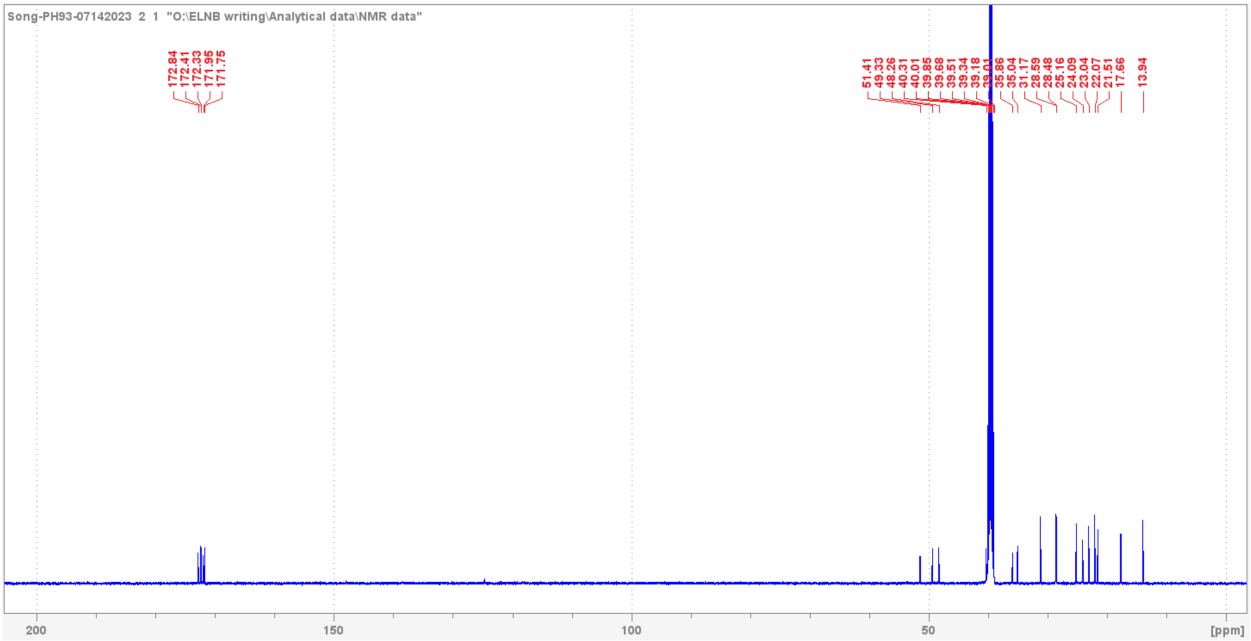

Figure S17: ^{13}C NMR of compound **11** in DMSO-d6.

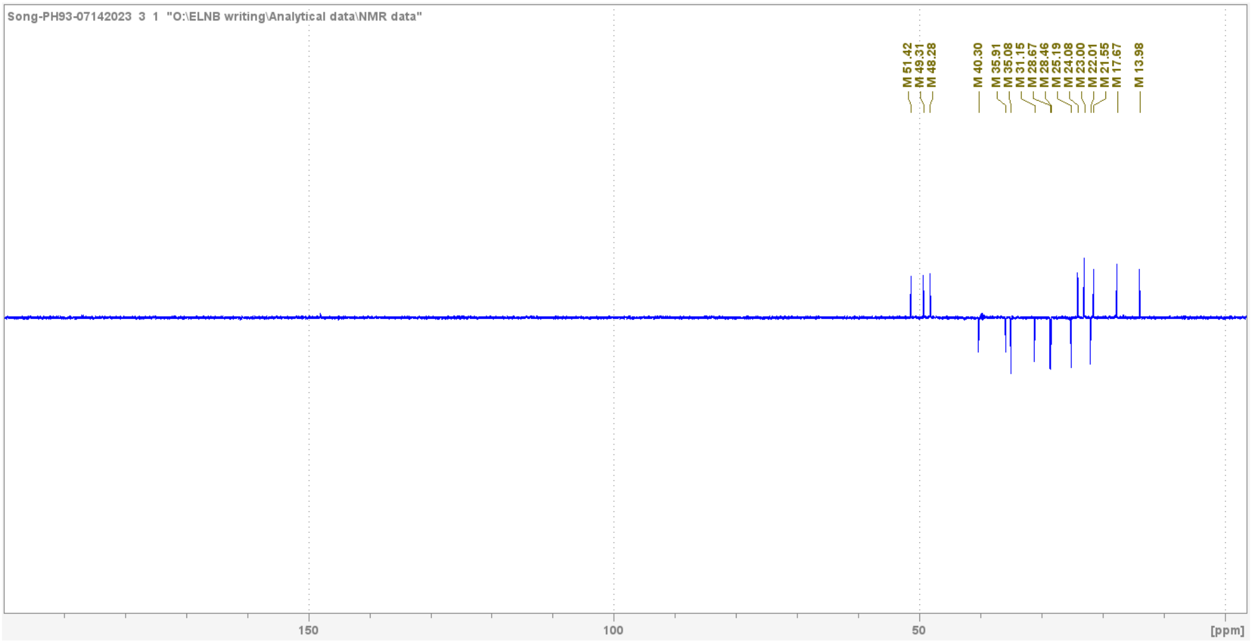

Figure S18: ^{13}C DEPT NMR of compound **11** in DMSO-d6.

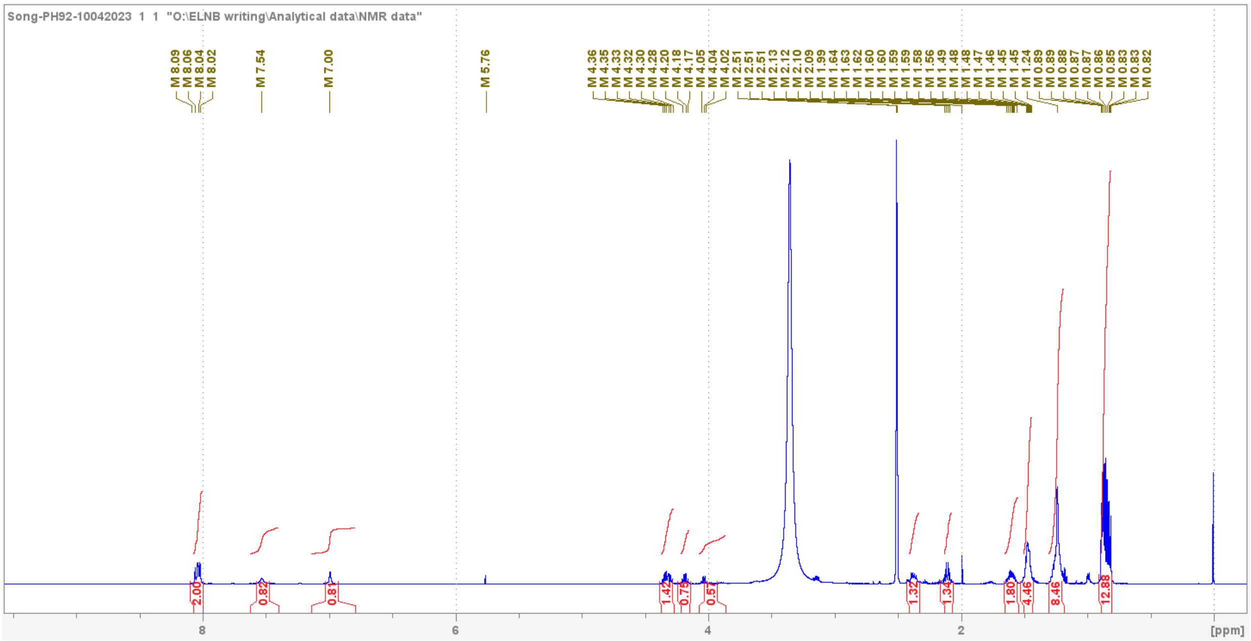

Figure S19: ^{1}H NMR of compound **12** in DMSO-d6.

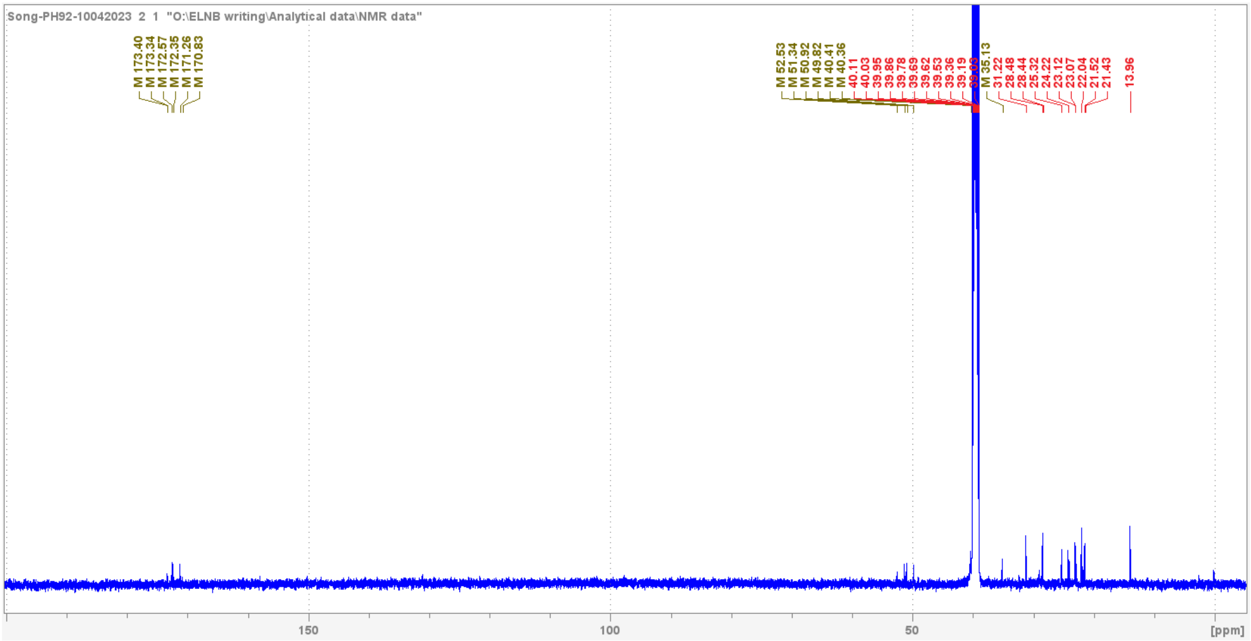

Figure S20: ^{13}C NMR of compound 1**2** in DMSO-d6.

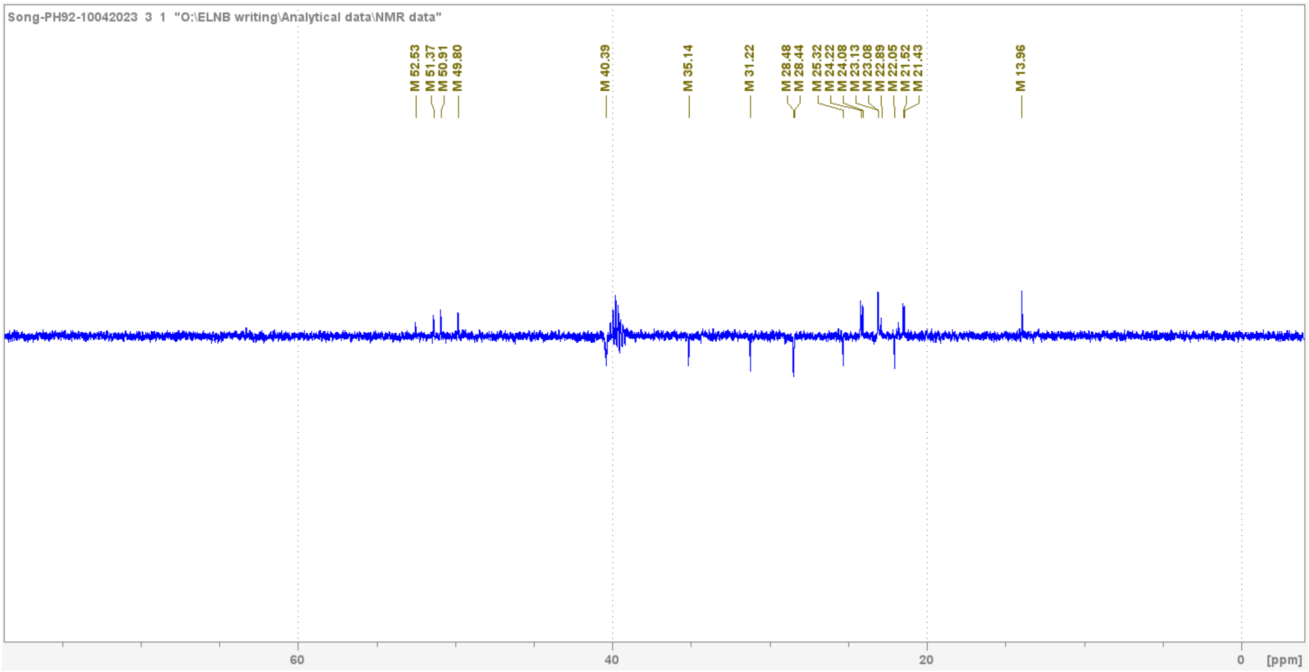

Figure S21: ^{13}C DEPT NMR of compound 1**2** (PH91) in DMSO-d6.

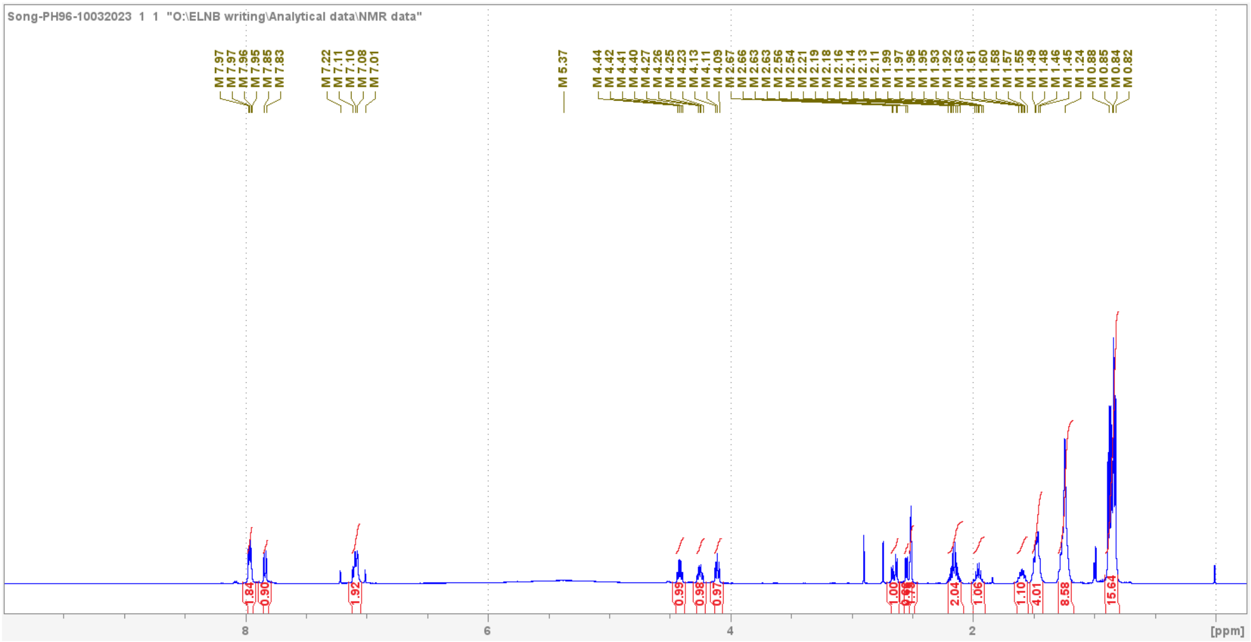

Figure S22: ^{1}H NMR of compound **13** in DMSO-d6.

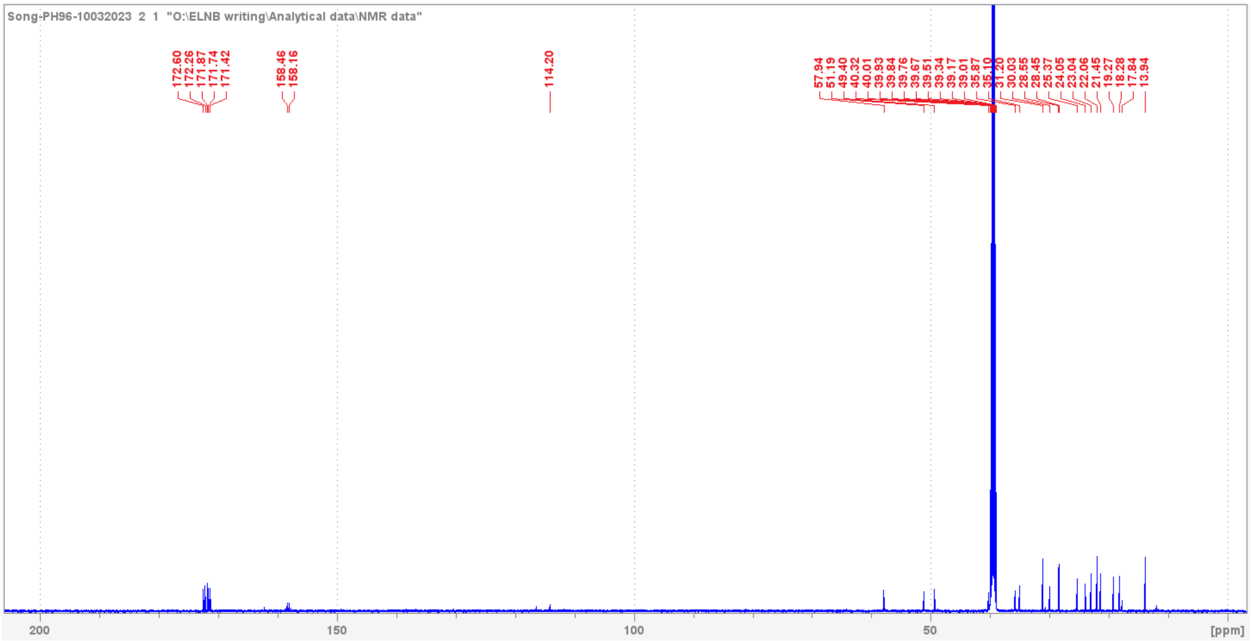

Figure S23: ^{13}C NMR of compound 1**3** in DMSO-d6.

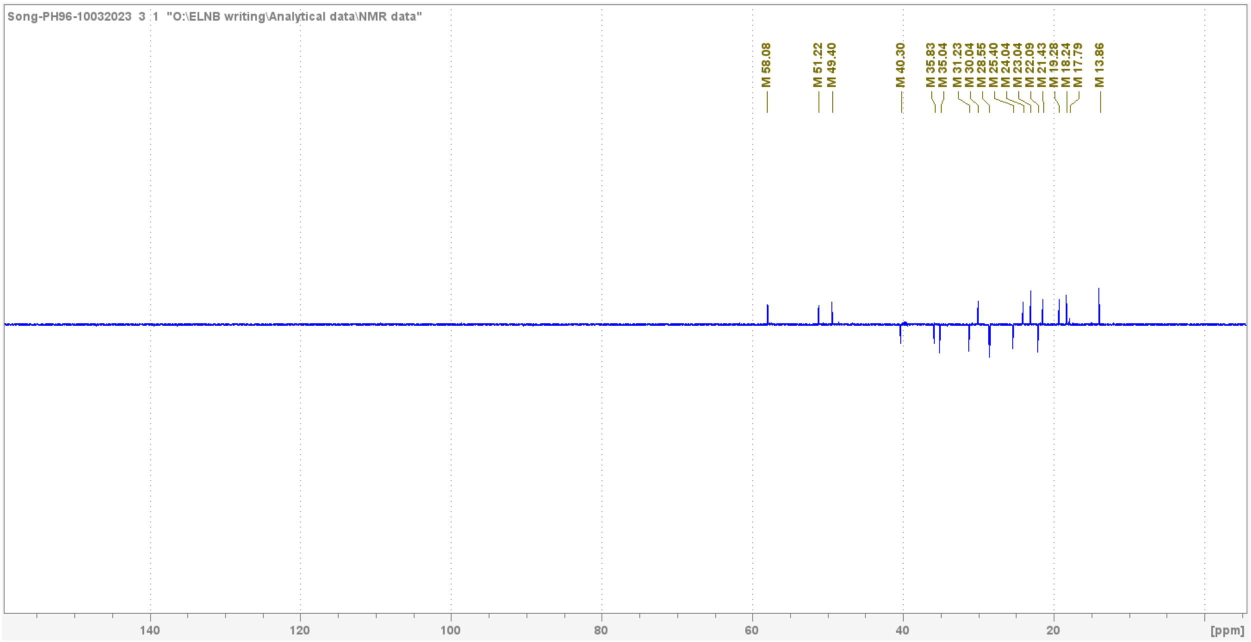

Figure S24: ^{13}C DEPT NMR of compound **13** in DMSO-d6.

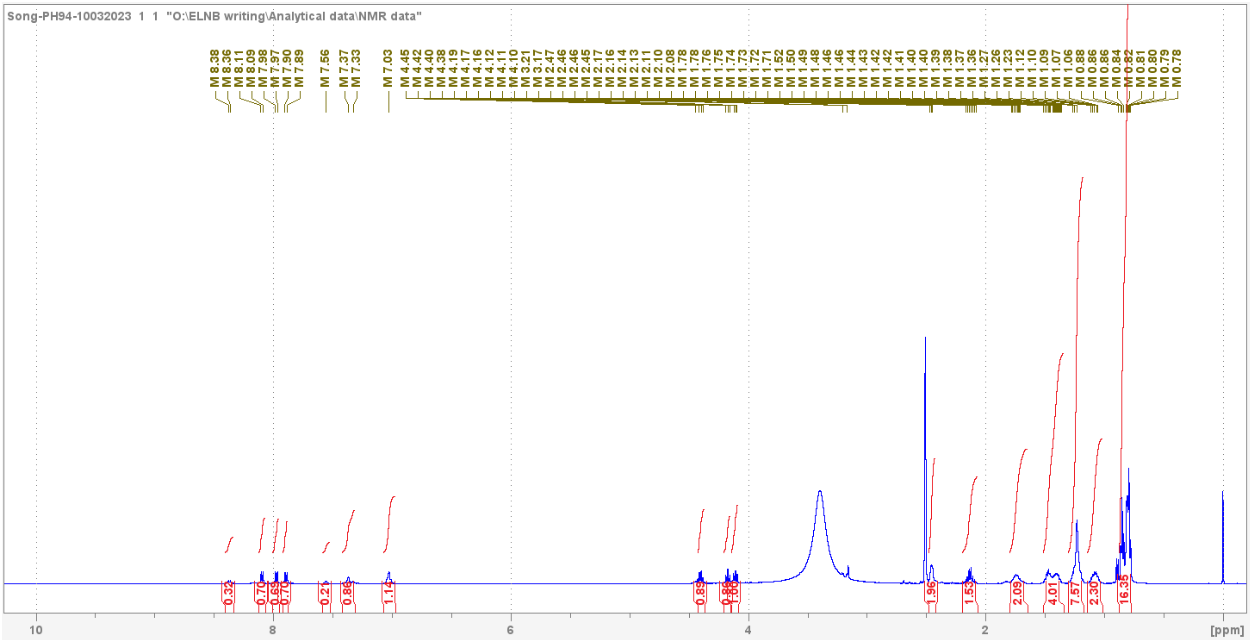

Figure S25: ^{1}H NMR of compound **14** in DMSO-d6.

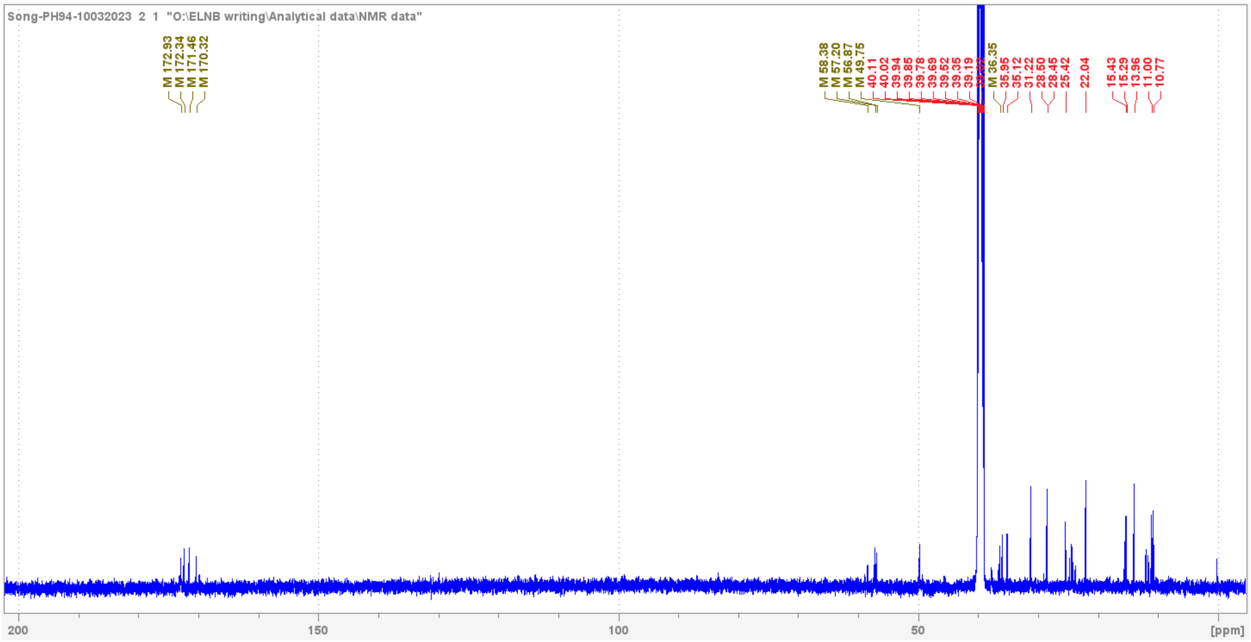

Figure S26: ^{13}C NMR of compound **14** in DMSO-d6.

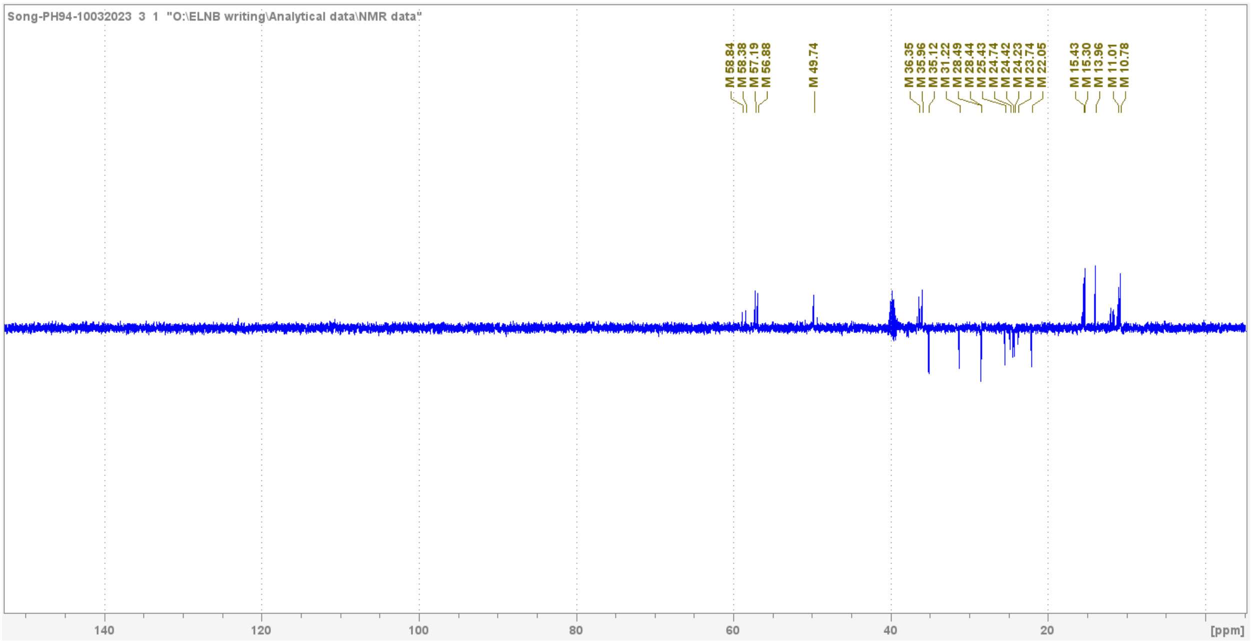

Figure S27: ^{13}C DEPT NMR of compound 1**4** in DMSO-d6.
